# Deformation of the nucleus by TGFβ1 via the remodeling of nuclear envelope and histone isoforms

**DOI:** 10.1186/s13072-021-00434-3

**Published:** 2022-01-04

**Authors:** Ya-Hui Chi, Wan-Ping Wang, Ming-Chun Hung, Gunn-Guang Liou, Jing-Ya Wang, Pen-Hsiu Grace Chao

**Affiliations:** 1 Institute of Biotechnology and Pharmaceutical Research, National Health Research Institutes, 35 Keyan Road, Zhunan, Miaoli County, 35053 Taiwan; 2 Graduate Institute of Biomedical Sciences, China Medical University, Taichung, 40402 Taiwan; 3 National Taiwan University College of Medicine, Taipei, 10051 Taiwan; 4 Department of Biomedical Engineering, School of Medicine and School of Engineering, National Taiwan University, Taipei, 10617 Taiwan

**Keywords:** Nuclear envelope, Nuclear lamina, Nuclear morphology, TGFβ1

## Abstract

The cause of nuclear shape abnormalities which are often seen in pre-neoplastic and malignant tissues is not clear. In this study we report that deformation of the nucleus can be induced by TGFβ1 stimulation in several cell lines including Huh7. In our results, the upregulated histone H3.3 expression downstream of SMAD signaling contributed to TGFβ1-induced nuclear deformation, a process of which requires incorporation of the nuclear envelope (NE) proteins lamin B1 and SUN1. During this process, the NE constitutively ruptured and reformed. Contrast to lamin B1 which was relatively stationary around the nucleus, the upregulated lamin A was highly mobile, clustering at the nuclear periphery and reintegrating into the nucleoplasm. The chromatin regions that lost NE coverage formed a supra-nucleosomal structure characterized by elevated histone H3K27me3 and histone H1, the formation of which depended on the presence of lamin A. These results provide evidence that shape of the nucleus can be modulated through TGFβ1-induced compositional changes in the chromatin and nuclear lamina.

**Supplementary Information:**

The online version contains supplementary material available at 10.1186/s13072-021-00434-3.

## Introduction

The cell nucleus is a double membrane-enclosed organelle. Most nuclei appear spheroid or ellipsoid; however, the shape can vary from trilobed in human neutrophils to dumbbell-shaped in some white blood cells. Nuclear atypia which refers to abnormally shaped cell nuclei is a term used in cytopathology, and is considered a significant indicator of malignancy [1]. The morphology of the cell nucleus is also a key indicator of the disease state and prognosis of progeria, neurodegenerative diseases and virus infection [2–7]. Changes in nuclear shape have been linked to chromatin reorganization and gene expression [5, 8]; however, the molecular signaling underlying the variations in nuclear morphology has yet to be elucidated.

The nuclear lamina beneath the inner nuclear membrane is a meshwork of type V intermediate filament proteins consisting primarily of A- and B-type lamins [9]. The expression of B-type lamins is relatively constant among tissues, whereas the abundance of lamin A vary systematically by as much 30-fold between soft and stiff tissue. High lamin A levels can physically stabilize the nucleus against stress and thereby protect the nuclear lamina and chromatin. It is suggested that the mechanical signals transmitted from the extracellular environment to the nucleus mediated by the cytoskeleton may fine tune the lamin A:B for cell-specific gene expression [10]. Abnormalities in the nuclear lamina are hallmarks of many human diseases [11]. Different types of lamina abnormality, such as herniations, honeycomb-like structures, and irregular staining, have been observed in primary dermal fibroblasts derived from *LMNA*-variant carriers [12]. These findings indicate that the level and composition of nuclear lamins in different tissues must be fine-tuned in a manner that prevents rupturing of the NE without constraining migration [13].

Nuclear lamins interact closely with chromatin in regions referred to as lamina-associated domains (LADs), which are formed by heterochromatin that have a low gene frequency, are transcriptionally silent, and enriched with repressive histone marks, H3K9me2/3 [14]. In one study involving the characterization of *Drosophila melanogaster* genome at the nuclear lamina, gene expression and active histone marks were shown to correlate with reduced lamina binding [15]. Similarly, lamina-associated-polypeptide 2 (LAP2) isoforms bind the histone deacetylase HDAC3, resulting in deacetylation of histone H4 and transcriptionally repressive activity [16]. Lamin B receptor (LBR) forms a tight complex with heterochromatin protein HP1 and histones H3/H4, which possess predominantly heterochromatic epigenetic marks [17]. On the other hand, it has been shown that lamin B1 associates with actively expressed and open euchromatin regions during epithelial-to-mesenchymal transition (EMT), resulting in the formation of dynamic euchromatin lamin B1-associated domains (eLADs) [18]. Overall, these evidences suggest that nuclear lamins may alternate their behavior by associating with active or repressive chromatin regions in response to extracellular signaling.

The transforming growth factor-β (TGFβ) superfamily, including TGFβ, Nodal, bone morphogenetic proteins (BMPs), play important roles in development, tissue homeostasis, cell proliferation and apoptosis. TGFβ signaling has been implicated in diseases, such as asthma, diabetes, fibrotic diseases, Marfan syndrome, Loeys–Dietz syndrome and cancer [19]. TGFβ family members relay their signals through binding to heterotetrameric complexes of type I and type II dual specificity kinase receptors. Of them, TGFβ1 binds to the type II receptor which recruits and phosphorylates the type I receptor to phosphorylate members of the receptor-activated (R)-Smad family, such as SMAD2 and SMAD3. The activated (R)-Smad then forms trimeric complexes with the common mediator SMAD4, which is translocated to the nucleus, where they cooperate with other transcription factors, histone modification coactivators/corepressors to regulate the expression of specific genes [20]. In premalignant stages of cancer, TGFβ1 acts as a tumor suppressor by inhibiting proliferation and inducing apoptosis in epithelial cells. On the other hand, in later stages of cancer development, TGFβ1 increases the migratory and invasive capacity of cancer cells by inducing EMT [21].

Cancer cells utilize EMT in the migration from their epithelial cell community and integration into tissue at remote locations (i.e., distant metastasis). This switch in cell differentiation and behavior is mediated by changes in cell morphology as well as post-transcriptional and post-translational gene regulation [20, 21]. Whereas changes in cell shape are linked to local gradients in signaling molecules for the subsequent cell activities [22], the means by which the nuclear shape is regulated in response to extracellular signaling remains unclear. In this study, we discovered that shape of the nucleus became highly deformed under the treatment of TGFβ1. The nuclear envelope (NE) proteins SUN1 and the B-type lamin, and the SMAD-downstream upregulation of a histone H3 variant H3.3, are required for this process. Whereas the A-type lamin is dispensable for the TGFβ1-induced nuclear deformation, it is recruited to enclose the NE after the rupture, as well as the clustering of H3K27me3 and histone H1. These results provide evidence that nuclear shape is linked to TGFβ1 signaling involved in the compositional remodeling of the nuclear lamina, core histones, and linker histones.

## Results

### Deformation of nuclear morphology induced by TGFβ1

**Fig. 1:**
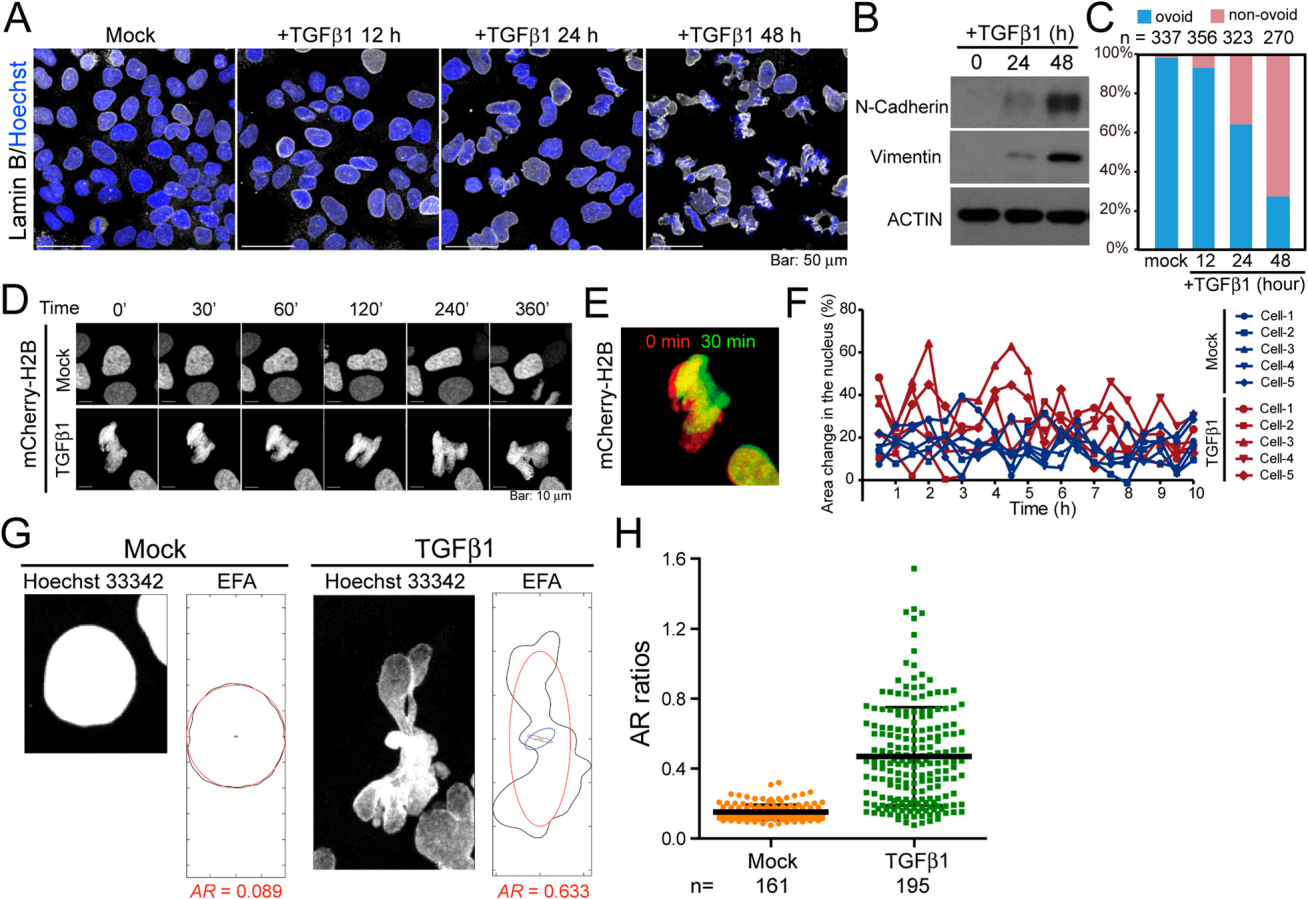
TGFβ1 induces nuclear deformation. **A** Confocal images of Huh7 cells with mock or with TGFβ1 treatment for 12 h, 24 h and 48 h. Cells were immunofluorescent stained with goat anti-lamin B (white). Nuclei were counterstained with Hoechst 33342 (blue). **B** Western blot analysis for the expression of mesenchymal markers N-Cadherin and Vimentin in Huh7 cells harvested after 0, 24 h, and 48 h of TGFβ1 treatment. ACTIN is a loading control. **C** Quantification of mock- and TGFβ1-treated cells, presenting as ovoid or non-ovoid, as shown in (**A**). Nuclei with more than two > 240° invaginations were identified as non-ovoid [40]. Number of cells quantified under each experiment condition was denoted. **D** Time-lapse confocal microscopic images of mCherry-tagged histone H2B (mCherry-H2B) in mock- and TGFβ1-treated Huh7 cells. In addition, see Additional file 2: Movie S1 and Additional file 3: Movie S2 (started to record after 24 h of TGFβ1 treatment). **E**, **F** Quantification of morphological changes (area in green divided by area in yellow + green) in the area of the nucleus in mock- and TGFβ1-treated cells every 30 min. Five cells were quantified under each condition. Percent area change is 15.72 ± 0.78 in mock-treated and 24.66 ± 1.278 in TGFβ1-treated cells. *P* < 0.0001, *t* test. **G** Confocal images of nuclei stained with Hoechst 33342 (white, left images in each panel) and representative illustration of ellipse generation (right schemes in each panel, outlined by circles) by EFA to approximate the shape of the nucleus. The elliptic ARs increased with the curvature of the nucleus. **H** Quantification of ARs in Huh7 cells with mock or TGFβ1 treatment for 48 h. Each dot represents one cell. More than 160 cells were quantified under each condition. *P* < 0.0001, *t* test

Transforming growth factor beta 1 (TGFβ1) is a pleotropic cytokine essential to a variety of cellular functions, including EMT. In addition to the dramatic phenotypic change, such as loss cell–cell adhesion and profound reorganization of the cytoskeleton (Additional file 1: Fig. S1A), we serendipitously discovered that the nuclear morphology of Huh7 hepatocellular carcinoma cells became abnormally shaped when treated with TGFβ1 (Fig. 1A), concomitant with increases in the expression of mesenchymal markers N-Cadherin and Vimentin (Fig. 1B). The nuclear morphology gradually deformed over time, with more than 70% of the nuclei becoming non-ovoid after two days of TGFβ1 treatment (Fig. 1A, C and Additional file 1: Fig. S1B). Live-cell imaging of fluorescent histone H2B revealed that shape of the nucleus in TGFβ1-treated cells progressively deformed from normal, and was more dynamic than in mock-treated cells (Fig. 1D, Additional file 2: Movie S1 and Additional file 3: Movie S2). Measured at intervals of 30 min post-treatment, the percent change was roughly 60% higher in TGFβ1-treated cells than in mock-treated cells (Fig. 1E, F). Using elliptic Fourier analysis (EFA) to compute elliptic axial ratios (ARs) describing the nuclear shape [23], we identified a drastic increase in shape abnormalities in TGFβ1-treated nuclei (Fig. 1G, H). TGFβ1-induced nuclear shape aberrations were also observed in RD (human rhabdomyosarcoma), NMuMG (mouse mammary gland epithelial cell), and HT-1080 (human fibrosarcoma) cells (Additional file 1: Fig. S1C, D). These results revealed that morphology of the nucleus becomes highly deformed under TGFβ1 stimulation, an observation similar to nuclear atypia occurs in malignant tissues.

### Nuclear envelope (NE) proteins differ in their contributions to TGFβ1-induced nuclear deformation

**Fig. 2:**
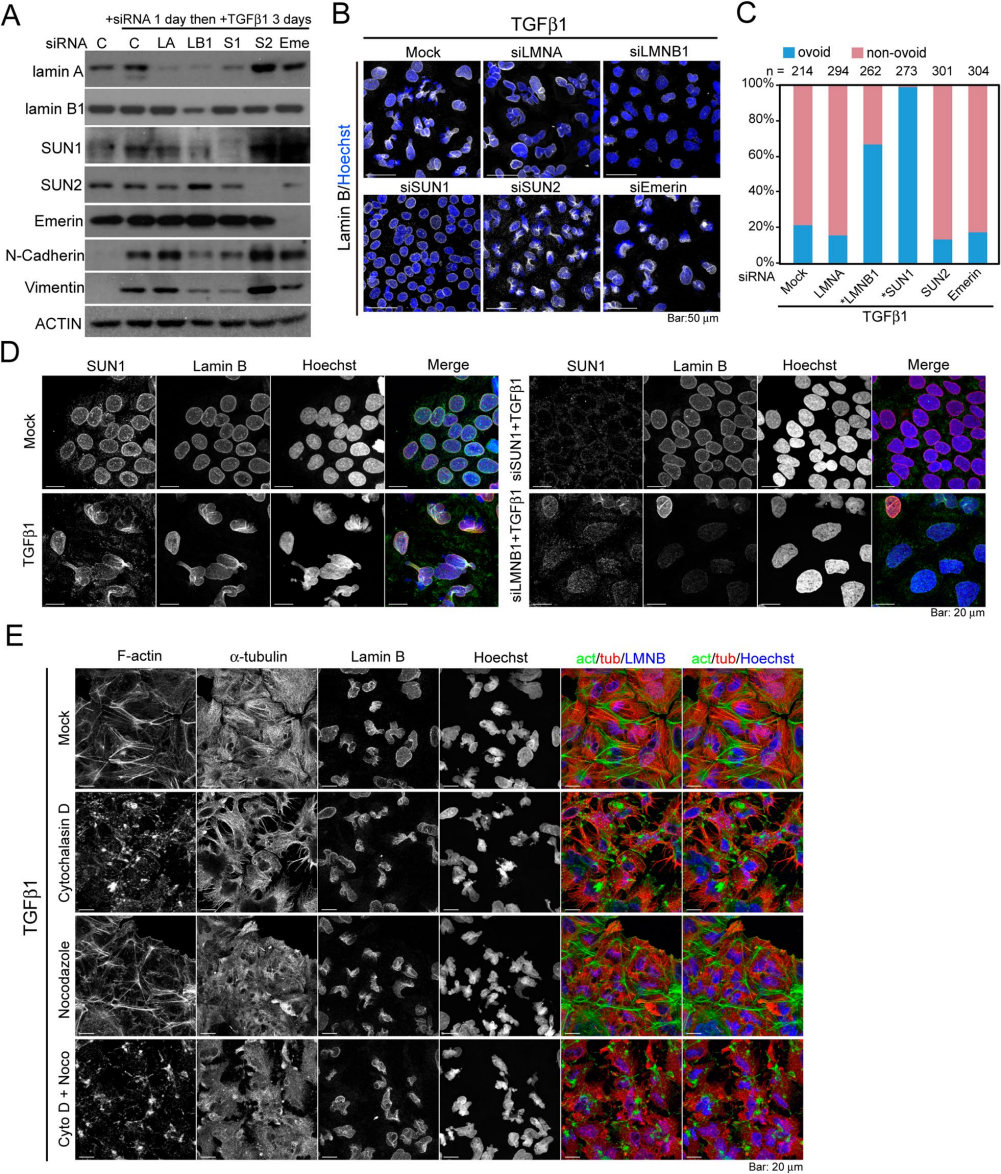
SUN1 and lamin B1, but not lamin A, contribute to TGFβ1-induced nuclear deformation. **A** Immunoblotting results of the indicated proteins in Huh7 cells transfected using the indicated siRNAs for 24 h then treated with 10 ng/mL TGFβ1 for 3 days. C, control; LA, lamin A; LB1, lamin B1; S1, SUN1; S2, SUN2; Eme, Emerin. ACTIN was used as a loading control. **B** Confocal images of cells transfected with the indicated siRNAs for 24 h, followed by TGFβ1 treatment for 48 h. Cells were immunofluorescent stained with lamin B (white) and the nuclei were counter stained with Hoechst 33342 (blue). **C** Categorization of the nuclear shape in cells treated using the methods in (**B**). Number of cells quantified under each experiment condition was denoted. *P < 0.0001, Fisher’s exact test. **D** Confocal images of cells transfected with siRNAs against SUN1 or lamin B1 for 24 h, followed by TGFβ1 treatment for 48 h. Cells were immunofluorescent stained with rabbit anti-SUN1 and goat anti-lamin B. The nuclei were counter stained with Hoechst 33342. **E** Confocal images of cells with mock or TGFβ1 treatment for 48 h, followed by 1 μM treatment of cytochalasin D and/or nocodazole for 30 min. Cells were immunofluorescent stained with phalloidin (to denote F-Actin), mouse anti-α-tubulin and goat anti-lamin B. Nuclei were counterstained with Hoechst 33342. Act, F-actin; tub, α-tubulin, LMNB, lamin B. All images are the sum of z-stacks

The nuclear lamina provides mechanical support to the nucleus via interactions with the LINC (linker of nucleoskeleton and cytoskeleton) complex comprising SUN (Sad1 and UNC84)-domain proteins and proteins that contain spectrin repeats [24, 25]. Thus, we sought to determine whether nuclear lamins and/or inner nuclear membrane (INM) proteins participate in TGFβ1-induced nuclear deformation. Knocking down lamin B1 or SUN1 using siRNAs modestly reduced the expression of mesenchymal markers N-Cadherin and Vimentin in cells stimulated by TGFβ1 to undergo EMT, and the nuclei remained ovoid-like. Conversely, the depletion of lamin A, SUN2, or Emerin had no effect on Vimentin expression or TGFβ1-elicited aberrancies in nuclear morphology (Fig. 2A–C). On the other hand, knocking down lamin B1 or SUN1 2 days after TGFβ1 treatment abolished the induced deformation of the nucleus (Additional file 1: Fig. S2A–C); the positive staining of Vimentin in the lamin B1- or SUN1-knockdown cells indicated the occurrence of EMT (Additional file 1: Fig. S2D). Consistent with the Western blot result, we noted that the depletion of lamin B1 reduced the level of SUN1, whereas the depletion of SUN1 had no effect on the level of lamin B1 (Fig. 2D). These results indicate that the mechanical forces transmitting through SUN1 and lamin B1 contribute to TGFβ1-induced changes in nuclear shape, regardless of the occurrence of EMT. Due to the dependence of SUN1 level on lamin B1, it is likely that SUN1 plays a major role in the TGFβ1-elicited nuclear shape abnormalities, and the lamin B1-mediated phenotype could be a secondary effect.

SUN1 is an inner nuclear membrane protein which links the nucleoskeleton and cytoskeleton. Therefore, knocking down SUN1 would decouple the NE from the cytoskeleton, as well as releasing the NE from the chromatin. To determine if the mechanical support from the cytoskeleton is responsible for the nuclear shape abnormalities, we followed the shape of the nucleus after the treatment of cytochalasin D and/or nocodazole which, respectively, disrupt polymerization of F-actin and microtubules (Fig. 2E). These results indicate that depolymerization of F-action and/or microtubules failed to restore the nuclear shape, suggesting that the tension within the nucleus should play a major role for the TGFβ1-induced nuclear deformation.

### Differential mobility of lamin A and lamin B1 during TGFβ1-induced nuclear deformation

**Fig. 3:**
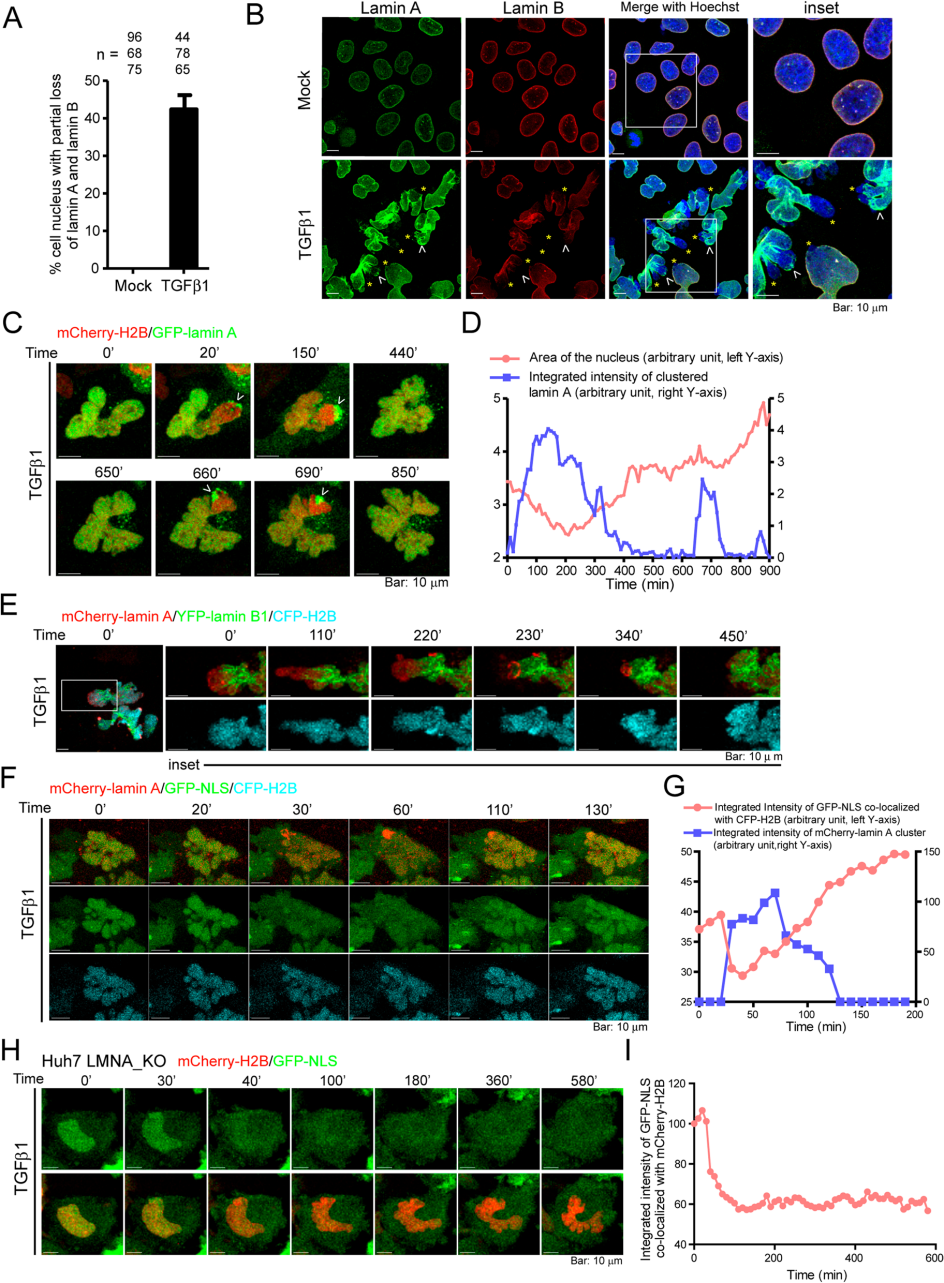
TGFβ1 induces rupturing and reformation of the NE. **A** Quantification of mock- and TGFβ1-treated (for 48 h) nuclei showing partial loss of lamin A and lamin B. Results were averaged from experiments conducted in triplicate. **B** Confocal images of mock- and TGFβ1-treated Huh7 cells for 48 h. Cells were immunofluorescent stained using mouse anti-lamin A (green) and goat anti-lamin B (red) antibodies. Nuclei were counterstained with Hoechst 33342 (blue). The yellow star indicates the NE stained negative for both lamin A and lamin B. The white arrow heads indicate the NE stained positive for lamin A and negative for lamin B. Insets: enlarged images indicated by white squares. **C** Time-lapse imaging of GFP-Lamin A (green) and mCherry-H2B (red) under TGFβ1 treatment. The labeled timepoints are relative to the initial image, rather than the time after TGFβ1 addition. The white arrow head denotes GFP-lamin A clusters. See also Additional file 4: Movie S3 (started to record after 24 h of TGFβ1 treatment). **D** Quantification of nucleus area (denoted by mCherry-H2B, peach) and integrated intensity of GFP-lamin A cluster (medium blue) with time shown in (**C**) and Additional file 4: Movie S3. **E** Time-lapse imaging of mCherry-lamin A (red), YFP-lamin B1 (green), and CFP-H2B (cyan) in Huh7 cells treated with TGFβ1. See also Additional file 5: Movie S4A, Additional file 6: Movie S4B (started to record after 24 h of TGFβ1 treatment). Insets: enlarged images showing the region outlined in the white square. **F** Time-lapse imaging of mCherry-lamin A (red), nuclear-localizing GFP (GFP-NLS, green), and CFP-H2B (cyan) in Huh7 cells treated with TGFβ1. See also Additional file 7: Movie S5A, Additional file 8: Movie S5B (started to record after 24 h of TGFβ1 treatment). **G** Quantification of integrated intensity of GFP-NLS co-localized with CFP-H2B (peach) and integrated intensity of mCherry-lamin A cluster (medium blue) as shown in (**F**). **H** Time-lapse imaging of mCherry-H2B (red) and GFP-NLS (green) in LMNA_KO Huh7 cells treated with TGFβ1. See also Additional file 9: Movie S6 (started to record after 24 h of TGFβ1 treatment). **I** Quantification of integrated intensity of GFP-NLS co-localized with mCherry-H2B as shown in (**H**). All images are the sum of z-stacks

The immunofluorescence staining images revealed that a portion of the nucleus in cells treated with TGFβ1 partially lost coverage of both A- and B-type lamins (Fig. 3A, B, yellow stars in Fig. 3B). A closer examination of the images revealed that part of the nucleus was stained negative for lamin B but positive for lamin A, the immunofluorescence signal of which was significantly higher following TGFβ1 treatment (white arrow heads in Fig. 3B). Western blot analysis showed that TGFβ1 provoked a significant increase in the expression of lamin A/C, but not lamin B1 or other INM proteins (e.g., SUN1, SUN2 and Emerin; Additional file 1: Fig. S3A, B). In the presence of TGFβ1, SUN1 and FG domain-containing nuclear pore complex (NPC) proteins overlapped more with lamin B than lamin A (Additional file 1: Fig. S3C, D), whereas Emerin co-localized more with the clustered lamin A (Additional file 1: Fig. S3E).

We subsequently followed the localization of lamin A in real time. Lamin A, which was initially distributed homogenously within the nucleus, became partially disassembled and leaked into the cytoplasm (Fig. 3C, compare times 0’, 20’ and 150’, Additional file 4: Movie S3). Within 10 min after the rupture, lamin A clusters appeared at the junction between the nucleus and cytoplasm, and then redistributed homogeneously throughout the nucleus (Fig. 3C, compare times 150’ and 440’). During this process, the nucleus in each cell expanded and regressed in size multiple times (Fig. 3D and Additional file 4: Movie S3). Live-cell imaging also revealed that the chromatin region covered with lamin A and devoid of lamin B1 was more mobile than the chromatin region covered with both lamin A and lamin B1 (Fig. 3E and Additional file 5: Movie S4A, Additional file 6: Movie S4B).

Using a nuclear-localizing green fluorescent protein (i.e., GFP-NLS) to track the localization of nuclear-residing proteins during TGFβ1-induced nuclear deformation, it was found that GFP-NLS leaked into the cytoplasm at the time of lamin A disassembly (Fig. 3F and Additional file 7: Movie S5A, Additional file 8: Movie S5B). As the clustered lamin A re-integrated into the nucleus, GFP-NLS was gradually imported from the cytoplasm into the nucleus (Fig. 3F, G). We further created a *LMNA*-knockout cell line (i.e., LMNA_KO) using the CRISPR/Cas9 method to verify the role of lamin A in the nuclear deformation process (Additional file 1: Fig. S3F). Similar to the results obtained using RNAi (Fig. 2B), the nuclear morphology of LMNA_KO cells deformed after TGFβ1 treatment (Additional file 1: Fig. S3G); however, nuclear-localizing GFP did not shuttle back to the nucleus once leaked into the cytoplasm (Fig. 3H, I; Additional file 9: Movie S6), indicating that lamin A is dispensable to the rupture, but crucial to the integrity of the NE during reformation. Intermittent, non-lethal ruptures of the nuclear envelope have been observed in dermal fibroblasts derived from patients of laminopathies and in *Lmna* knockout mouse embryonic fibroblasts [26]. The TGFβ1-induced rupture and deformation of the NE observed here should be different from the NE rupture events in *Lmna*-deficient cells due to the intactness of the nuclear lamina prior to TGFβ1 stimulation.

### Upregulation of histone H3.3 downstream of SMAD signaling is required for TGFβ1-induced nuclear deformation

**Fig. 4:**
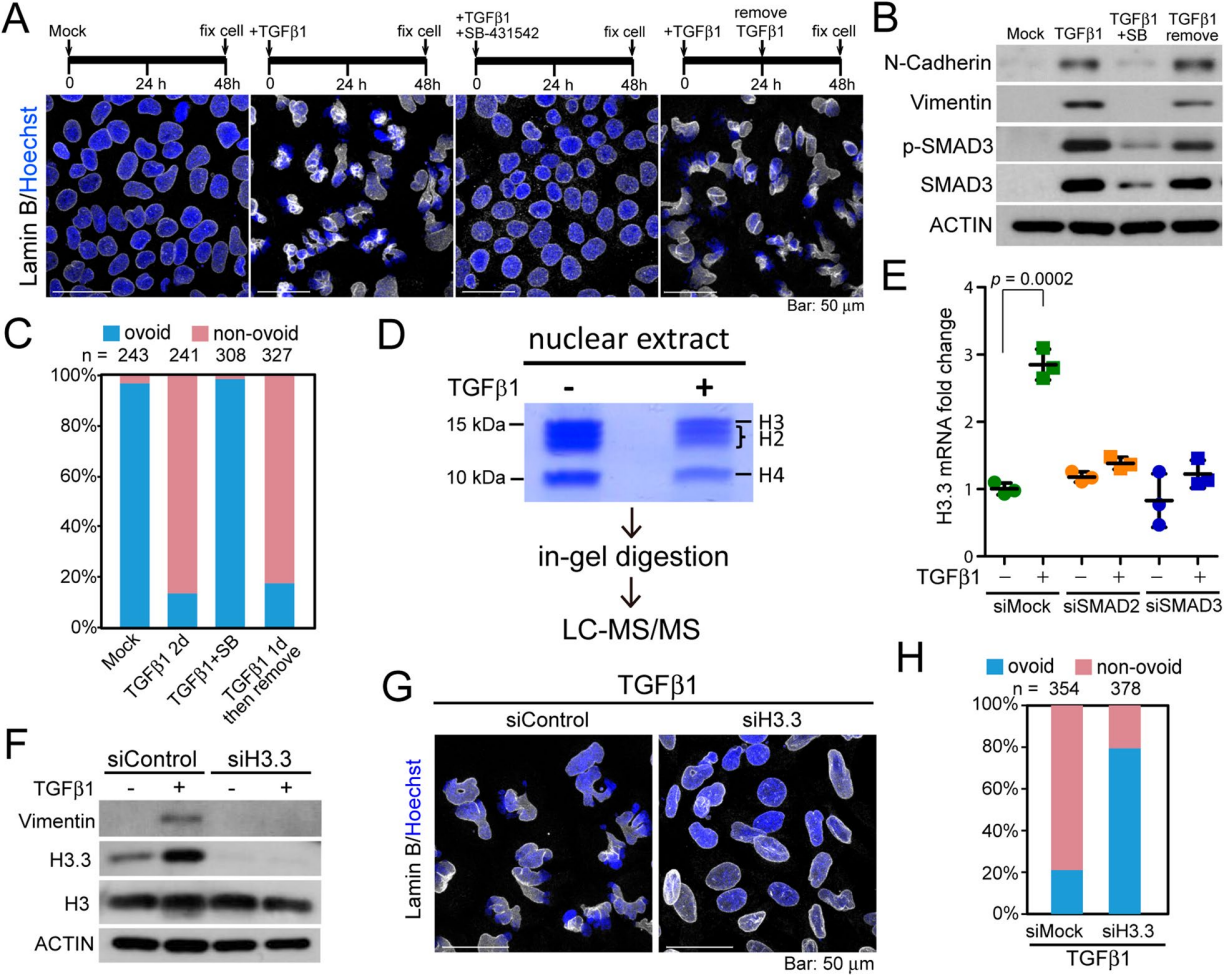
TGFβ1 provoked the transcription of histone H3.3, which contributed to nuclear deformation. **A** Morphology of the nucleus in Huh7 cells that underwent treatment in accordance with the schematic illustration presented above the confocal images. Cells were immunofluorescent stained using a lamin B antibody (white). Nuclei were counterstained with Hoechst 33342 (blue). **B** Western blot analysis for the expression of EMT markers in cells treated using the methods described in (**A**). SB, SB-431542 1 μM. **C** Categorization of nuclear shape in cells treated using the methods in (**A**). **D** SDS-PAGE stained with Coomassie blue for the nuclear extract from Huh7 cells without or with TGFβ1 treatment for 3 days. The histone H3 bands were excised, and subjected for in-gel digestion and LC–MS/MS analysis. **E** Quantitative RT-PCR for the relative mRNA expression levels of *H3.3* in mock- and TGFβ1-treated cells (for 72 h) pretreated with mock or SMAD2/SMAD3 siRNAs for 24 h. *P* value: *t* test. **F** Western blot analysis for the expression of Vimentin, H3.3, and H3 in Huh7 mock- or H3.3 siRNA-treated, and induced to undergo EMT by TGFβ1 for 72 h. ACTIN immunoblotting was used as a loading control. **G** Morphology of nucleus as indicated by immunofluorescence staining of lamin B (white) in control and H3.3-depleted Huh7 cells treated with TGFβ1 for 48 h. Nuclei were counterstained with Hoechst 33342 (blue). Images are the sum of z-stacks. Categorization of the nuclear shape (ovoid or non-ovoid) is summarized in (**H**)

We sought to determine whether phosphorylation of the receptor-activated (R)-SMAD family is required for TGFβ1-induced nuclear deformation. We found that shape of the nucleus remained ovoid in the presence of SB-431542, a selective inhibitor of TGF-βRI blocking phosphorylation of the SMAD complex. Removal of TGFβ1 at 24 h after the addition of TGFβ1 had no effect on the tendency toward nuclear deformation (Fig. 4A–C). Knocking down SMAD2 or SMAD3 using siRNAs reduced the extent of nuclear morphology alteration (Additional file 1: Fig. S4A–C), whereas overexpressing SMAD2 (tagged with HA) was sufficient to trigger deformation of the nucleus in the absence of TGFβ1 (Additional file 1: Fig. S4D, denoted by white arrow head). These results indicate that SMAD-downstream signaling contributes to TGFβ1-elicited nuclear deformation; removal of extracellular TGFβ1 failed to restore the nuclear shape once the process is initiated.

To ask if the physical presence of SMAD2/3, or their mediated transcription, is responsible for TGFβ1-induced nuclear deformation, we determined the nuclear shape in Huh7 cells co-treated with TGFβ1 and Actinomycin D, a DNA intercalators which blocks the progression of RNA polymerases [27]. As a result, the nuclear shape remained ovoid in cells co-treated with TGFβ1 and Actinomycin D, suggesting that transcription is required for the misshapen nuclei (Additional file 1: Fig. S4E).

The rigidity of chromatin is closely associated with the epigenetic status of the histone tails [28, 29]. Thus, we adopted a proteomic strategy to identify novel epigenetic modifications of histone H3 under TGFβ1 treatment (Fig. 4D). No significant differential epigenetic modification was detected (Additional file 1: Table S1). Rather, our LC–MS/MS results revealed that the protein level of histone H3.3 increased by roughly 2.6-fold following the treatment with TGFβ1 (Additional file 1: Table S2). This observation was verified by Western blot analysis and qRT-PCR of *H3-3A* (i.e., the H3.3 gene, Fig. 4E, F). Knocking down *H3-3A* using siRNAs abolished both TGFβ1-induced EMT (as indicated by Vimentin expression) and nuclear deformation (Fig. 4F–H). Furthermore, depleting SMAD2 or SMAD3 reduced the transcription of *H3-3A* (Fig. 4E). These findings indicate that the deformation of nuclear morphology induced by TGFβ1 is a SMAD-downstream event following the upregulation of H3.3*.*

### Enrichment of histone H1 and H3K27me3 at chromatin regions that lost NE coverage

**Fig. 5:**
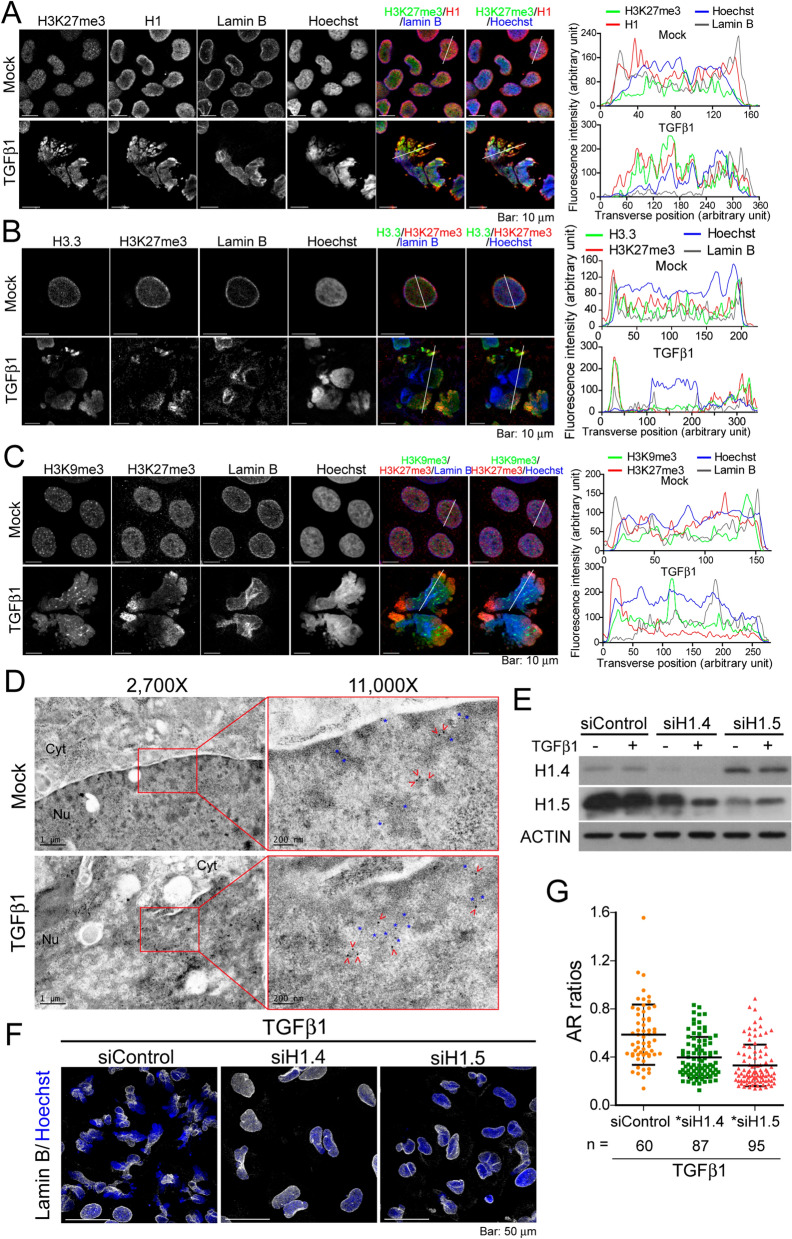
Chromatin status associated with NE rupture. **A** Confocal images of mock- and TGFβ1-treated cells immunofluorescence stained using H3K27me3, H1, and lamin B antibodies. Nuclei were counterstained with Hoechst 33342. **B** Confocal images of mock- and TGFβ1-treated cells immunofluorescent stained using H3.3, H3K27me3 and lamin B antibodies. **C** Relative distribution of the epigenetic marks H3K27me3, H3K9me3 and lamin B in mock- and TGFβ1-treated cells for 48 h. **A**–**C** Single slice images. Transverse intensity line scans along the white lines in the corresponding cell images are presented on the right. **D** TEM images of mock- and TGFβ1-treated cells stained for H3K27me3 (12 nm gold–IgG, blue star), histone H1 (18 nm gold–IgG, red arrow head), and lamin B (6 nm gold–IgG, not denoted). Images are shown under 2700× and 11,000× magnification. **E** Western blot analysis indicating the knocking down efficiency of H1.4 and H1.5 by siRNAs in Huh7 cells, followed by mock- or TGFβ1-treatment for 48 h. **F** Morphology of the nucleus in Huh7 cells depleted for H1.4 or H1.5, followed by TGFβ1 treatment for 48 h. Cells were immunofluorescent stained with lamin B (white). Nuclei were counterstained with Hoechst 33342 (blue). Images are the sum of z-stacks. **G** Quantification of elliptic ARs of the cells treated using the methods in (**F**). *, *P* < 0.0001, *t* test

We next sought to identify the epigenetic modification(s) of histones associated with NE rupture. Using antibodies that recognize specific epigenetic modifications of histones, we discovered that the immunofluorescence signals of H3K27me3 and H1 were well correlated, and enhanced in chromatin regions that had lost lamin B upon TGFβ1 stimulation (Fig. 5A and Additional file 1: Fig. S5A). Immunofluorescence staining revealed that the intensity of H3K27me3 was strongly correlated with H3.3 localization in cells subjected to TGFβ1 treatment (Fig. 5B). The distribution of H3K27me3 was shown not to overlap with H3K9me3, both of which are markers for heterochromatin (Fig. 5C) [8]. In transmission electron microscopic (TEM) images, heterochromatin generally appears as small, darkly stained, irregular particles scattered throughout the nucleus or accumulated adjacent to the NE. We used immunogold labeling to characterize the ultrastructural organization of subcellular features of the chromatin associated with H3K27me3 enrichment at nanoscale. The chromatin regions labeled with H1 (indicated by the red arrow head) and H3K27me3 (indicated by the blue star) appeared less dark in TGFβ1-treated cells than in untreated cells, which is indicative of lower chromatin packing density (Fig. 5D).

The monoclonal histone H1 antibody in Fig. 5A recognized the histone H1 variants H1.4 and H1.5. When using RNAi, it was found that the depletion of either H1.4 or H1.5 ameliorated TGFβ1-induced nuclear deformation, and the NE remained intact (Fig. 5E, F). The nucleus of cells depleted for H1.4 or H1.5 did not present an ovoid morphology; however, the degree of deformation was lessened, as evidenced by the AR ratios (Fig. 5F, G). By contrast, the TGFβ1-induced nuclear morphology was unaffected by the depletion of H1.2 or H1.3 (Additional file 1: Fig. S5B, C). These results suggest that incorporation of specific variants of linker histone H1 occur prior to the NE rupture.

### Lamin A contributes to TGFβ1-induced clustering of histone H1 and H3K27me3

**Fig. 6:**
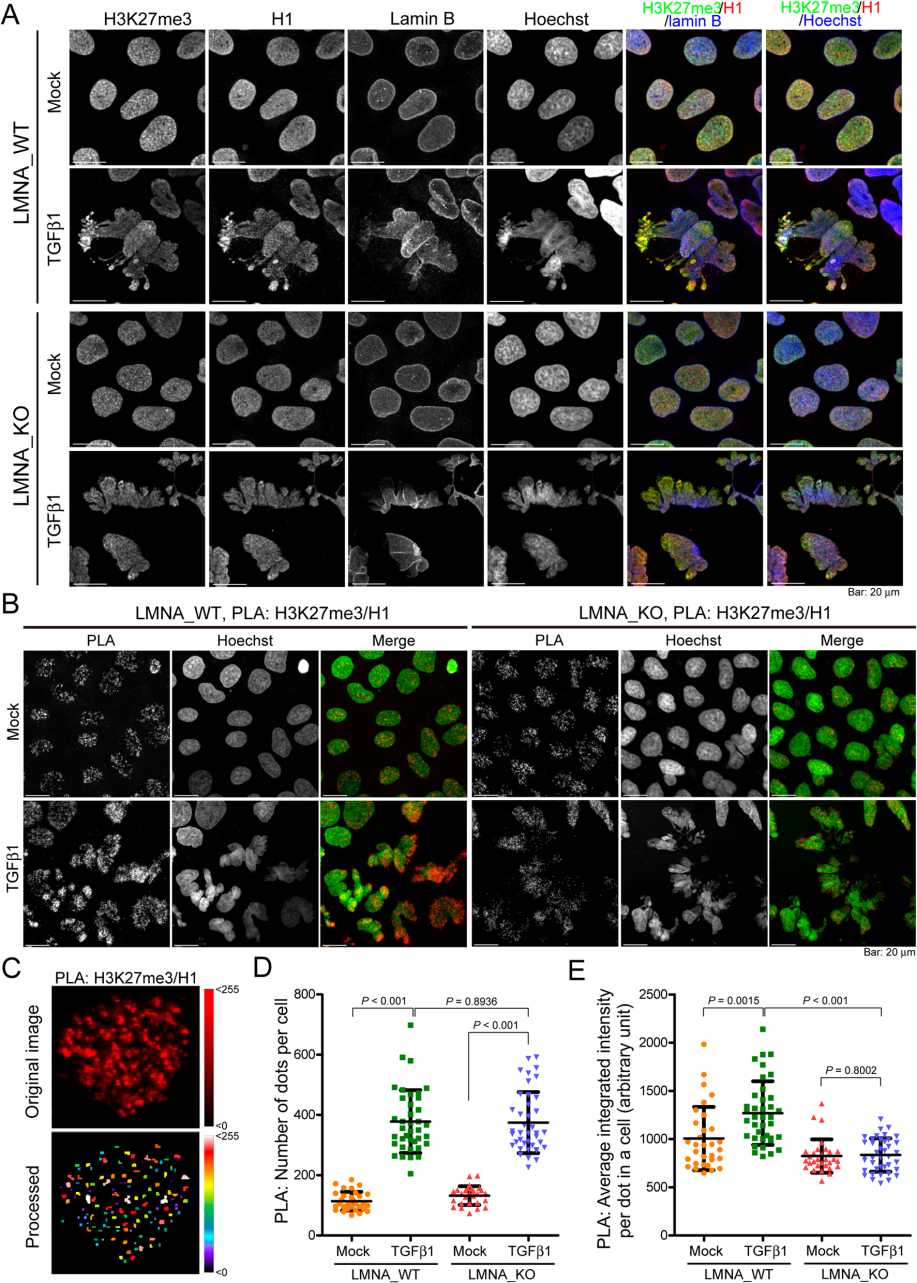
Depletion of *LMNA* reduced clustering of H3K27me3 and histone H1. **A** Representative confocal microscopy images of LMNA_WT and LMNA_KO Huh7 cells treated with/without TGFβ1 and immunostained using H3K27me3, H1, and lamin B antibodies, and Hoechst 33342. **B** Cells treated as described in (**A**) were subjected to PLA analysis. Each fluorescent dot represents the colocalization between H3K27me3 and histone H1. **C** Representative images showing the number and integrated intensity of the PLA fluorescent dots in a nucleus using MetaMorph^®^ software; (upper) original fluorescence image; (lower) processed image. The average intensity of each dot is color-coded according to the scale on the right. **D** Number of PLA dots in each cell. **E** Average integrated intensity of each dot in each cell. Cell number calculations in (**D**) and (**E**) were: LMNA_WT, *n* = 31; LMNA_WT + TGFβ1, *N* = 38; LMNA_KO, *n* = 30; LMNA_KO + TGFβ1, *N* = 40. All images are the sum of z-stacks

There have been reports of lamin A/C interacting with the Polycomb group (PcG) of proteins, such as EZH2, for their nuclear compartmentalization and transcriptional regulation [30, 31]. Therefore, we sought to determine whether lamin A is involved in the localization of H3K27me3 in Huh7 cells stimulated using TGFβ1. In LMNA_KO Huh7 cells, we did not observe significant clustering of H3K27me3 or histone H1 in the chromatin regions of TGFβ1-induced deformed nuclei that lost lamin B coverage (Fig. 6A). To determine whether *LMNA* depletion alters the association between H3K27me3 and histone H1, we used a proximity ligation assay (PLA), which permits the detection of transient interactions occurring between two proximal proteins separated by < 30 nm (Fig. 6B, C) [32]. Our results revealed that H3K27me3 was in close proximity with histone H1 in TGFβ1—as well as mock-treated cells, and the incidence of the associations increased by roughly 3.3-fold following TGFβ1 treatment (*P* < 0.001). In the presence of TGFβ1, there was no difference in the number of PLA dots in LMNA_KO cells and LMNA_WT cells (*P* = 0.8936); however, the average integrated intensity of each dot within a cell was significant lower (*P* < 0.001) in LMNA_KO than in LMNA_WT cells (Fig. 6D, E). Together with the observation in immunofluorescence staining and immunogold TEM images (Fig. 5A, D), these results suggest that lamin A is not essential to the association between H3K27me3 and histone H1, but rather contributes to the formation of a supra-nucleosomal structure enrich with H3K27me3 and histone H1 upon TGFβ1 stimulation.

## Discussion

**Fig. 7:**
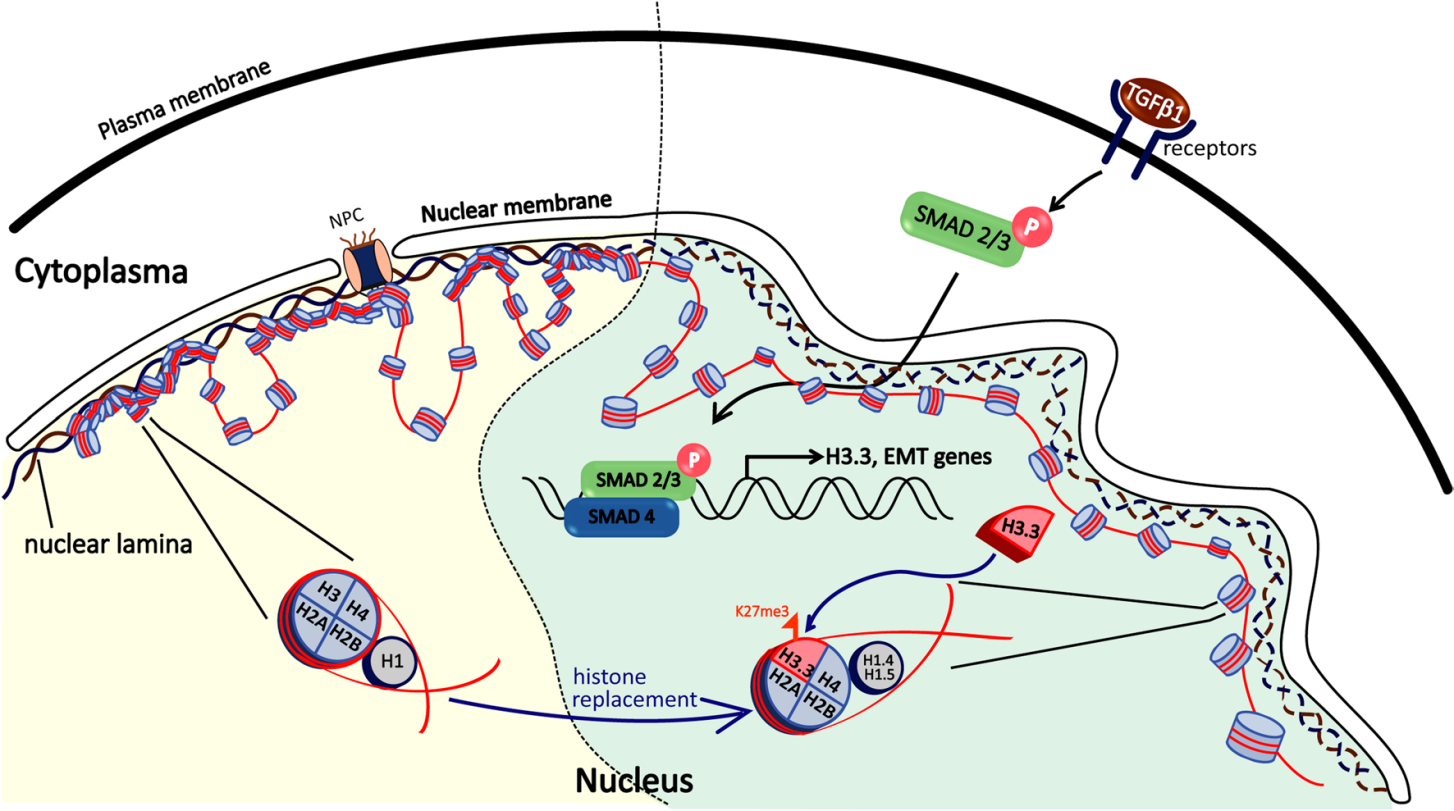
Schematics for the molecular events of the nuclear deformation induced by TGFβ1 stimulation. TGFβ1 treatment phosphorylates and activates the SMAD complex, thereby increasing transcription and the protein level of histone H3.3. The subsequent nucleosome incorporation of H3.3 may facilitate a dynamic chromatin environment that allows for further recruitment of histone H1.4/H1.5 variants, and the interaction with H3K27me3 for nuclear deformation and NE rupture

Abnormalities in nuclear morphology are hallmarks of many diseases, including progeria and cancer [33]. In the current study, we discovered that the multifunctional growth factor TGFβ1 alters the nuclear shape and induces NE rupture in a specific cell line subset. This cellular phenotype is a downstream signaling of SMAD2/3 phosphorylation, which requires the upregulation of histone H3.3 and the mechanical force link to nuclear lamin B1 and SUN1. We observed a strong correlation between the distribution of histones H1 and the H3K27me3 epigenetic mark in regions of chromatin that lost NE coverage, and this association is lamin A dependent. This led us to propose a biophysical model in which TGFβ1 signaling initially increases the expression of H3.3 for the subsequent transcription of EMT genes, followed by the incorporation of specific histone H1 variants and H3K27me3 epigenetic mark for nuclear deformation and NE rupture (Fig. 7).

The means by which the nucleus alters its morphology to allow cells to cross physical barriers and migrate through confined spaces has been investigated [34–36]. In studies on the migration of the nucleus through tight spaces, the incidence of NE rupture was shown to increase with cell confinement and the depletion of nuclear lamins [37, 38]. In those reports, opening of the NE allowed nuclear proteins to leak out of the nucleus and cytoplasmic proteins to leak in. In the current study, we also observed the nucleo-cytoplasmic shuttling of nuclear content upon constitutive rupture and reformation of the NE induced by TGFβ1 (Fig. 3D, E), implicating that the TGFβ1-initiated signal cascade may also play a role for cell migration through confined spaces.

Laminopathies that feature deformed nuclei are caused by mutations in *LMNA* [2]; however, the TGFβ1-induced nuclear deformation in this study was shown to depend on the presence of SUN1 and lamin B1, but not lamin A or SUN2 (Fig. 2B, C). Note that SUN1 co-localized with lamin B, but not with clustered lamin A (Additional file 1: Fig. S3C). These results suggest that there are inherent differences between SUN1 and SUN2 which are both components of the LINC complex, and between A- and B-type lamins in the lamina [39]. Whereas SUN1 and SUN2 have both been reported to interact with lamin A, they appear to have different roles in lamin A mutation-associated laminopathies including Emery–Dreifuss muscular dystrophy (EDMD) and Hutchinson–Gilford progeria syndrome (HGPS) [40, 41], as well as in mammalian development [42, 43]. For the nuclear lamins, in addition to have differential expression patterns in soft and stiff tissues [10], lamin B1 but not lamin A tends to be weak or absent at nuclear membrane protrusions (or blebs) [37, 39, 44–46]. We have observed differences in the expression levels and localization of lamin A and lamin B1 upon TGFβ1 stimulation (Fig. 3B and Additional file 1: Fig. S3). Interestingly, the chromatin regions covered with lamin A but not lamin B1 gained more mobility than did the chromatin regions covered with both lamin A and lamin B1 (Fig. 3E). Moreover, the depletion of lamin A prevented recovery of GFP-NLS in the nucleus upon TGFβ1-induced rupturing of the NE (Fig. 3H, I). These evidences suggest that the microdomains interacting with lamin B1 and SUN1 are crucial for TGFβ1-induced nuclear organization [39, 47, 48]. Lamin A and lamin B1 play different roles in regulating the nuclear shape; however, they are both required for closure of the NE following the rupture.

Lamin A levels directly or indirectly regulate many proteins involved in tissue-specific gene expression. TGFβ1 is a strong stimulator of collagen secretion [49], and lamin A responds to collagen levels, which scale with tissue stiffness [10]. In addition, lamin A/C modulates cellular responses to TGFβ1 signaling on collagen production [50]. In previous research, lamin A/C-rich NE blebs appeared condensed with transcriptionally active histone marks in lamin B2-deficient cells [39]. Lamin A/C has been discovered evolutionarily required for correct PcG-mediated nuclear compartmentalization and higher order structures [31]. In the current study we observed that *LMNA* depletion significantly reduced the TGFβ1-induced clustering of H3K27me3 and H1 (Fig. 6). It is likely that the lodging and dislodgement of lamin A within the chromatin may facilitate the formation and disassembly of the supra-nucleosomal structure associated with TGFβ1-induced transcriptional regulation.

In the current study, we found that the incorporation of histone H1, H3.3 and the H3K27me3 epigenetic mark was higher in regions of chromatin that herniated through the NE (Fig. 5A, B). Replacing canonical histones with histone variants in the nucleosome has previously been shown to modify chromatin structure and gene expression [51]. Specific incorporation of H3.3 into chromatin has been demonstrated both necessary and sufficient for the induction of aggressive traits that allow for metastasis formation [52]. The histone variant H3.3 maintains a decondensed chromatin state, and has been implicated in the balance between open and condensed chromatin, which is crucial to the fidelity of chromosome segregation during early mouse development [53]. H3.3 deposition has long been associated with gene activation; however, one genome-wide profiling study reported that H3.3 may facilitate a dynamic chromatin environment that allows for optimal PRC2 binding and activity, thereby promoting the establishment of a bivalent chromatin landscape in embryonic stem cells (ESCs) [54]. B-type lamins are closely associated with repressive chromatin [14]; therefore, our observation of H3.3 and H3K27me3 co-localization in chromatin regions devoid of nuclear lamin B indicates that TGFβ1 may initiate a cascade of gene transcription activities requiring the dislodgement of B-type lamins. Identifying the mechanism by which the nuclear lamins coordinate with histone variants for gene regulation in response to TGFβ1 will require further investigation [55, 56].

Members of the linker histone H1 family bind to nucleosomal core particles around DNA entry and exit sites, and stabilize both the nucleosome structure and higher order chromatin architecture [57]. H1 has long been seen as a general condenser of chromatin [58]; however, there is a growing body of evidence indicating that H1 has the potential to fine-tune transcription in a locus-specific manner [59, 60]. In this study, we discovered a subtype-specific (i.e., H1.4 and H1.5) requirement of histone H1 for the TGFβ1-induced nuclear deformation (Fig. 5E–G). The existence of multiple H1 subtypes and various posttranslational modifications adds to the complexity and challenges associated with studying this protein family [57, 61]. The collaboration of H1 subtype members with core histones in gene regulation would depend on the availability of antibodies that recognize specific subtypes of histone H1.

In summary, we discovered a novel phenotype involved in deformation of the nucleus under the effects of TGFβ1 signaling. The rupturing and reformation of the NE require multiple consecutive changes in the composition of the nuclear lamina as well as core and linker histones. These results reveal a molecular mechanism that renders the morphology of the nucleus responsive to TGFβ1 signaling, which plays a crucial role in tissue homeostasis and disease progression.

## Materials and methods

### Cell culture

Huh7 hepatocellular carcinoma cell line was sourced from JCRB cell bank (JCRB0403, Japan). Huh7, RD (CCL-136, ATCC, VA, USA) and NMuMG (CRL-1636, ATCC) cell lines were maintained in high glucose Dulbecco’s Modified Eagle Medium (DMEM, Thermo Fisher Scientific, Waltham, MA, USA) containing 10% fetal bovine serum (FBS, Hyclone, Logan, UT, USA), 2 mM l-glutamine and antibiotics. HT-1080 (CCL-121, ATCC) cell line was maintained in Eagle’s Minimum Essential Medium (MEM, Thermo Fisher Scientific) containing 10% FBS and supplemented with 2 mM l-glutamine, 1 mM sodium pyruvate, and antibiotics.

### Generation of *LMNA* null cell line

A human lamin A Double Nickase Plasmid set (sc-400039-NIC, Santa Cruz Biotechnology, Dallas, TX, USA) was used to generate *LMNA*-knockout (LMNA_KO) cells. The lamin A Double Nickase Plasmid set consists of a pair of plasmids each encoding GFP/puromycin selection markers, and guide RNA (gRNA) sequences offset by approximately 20 bp to allow for specific Cas9-mediated double nicking of *LMNA* genomic DNA. Huh7 cells were transfected with the lamin A Double Nickase Plasmid set using Lipofectamine™ 2000 (Thermo Fisher Scientific) transfection reagent. Two days after the transfection, top 5% GFP-positive cells were sorted using a BD Influx (BD Biosciences, San Jose, CA, USA) cell sorter, and individual cells were plated into 96-well plates. Expression of lamin A/C in each single clone were determined by Western blot analysis and immunofluorescence staining using a lamin A/C antibody (ab108595, Abcam).

### Antibodies and reagents

The manufacturers and dilutions of the antibodies used in Western blot analysis and immunofluorescence staining are listed in Additional file 1: Tables S3, S4, respectively. TGFβ1 was obtained from PeproTech (Rocky Hill, NJ, USA); the TGFβ type I receptor/ALK5 inhibitor SB-431542 was purchased from TOCRIS (Bristol, UK); cytochalasin D was obtained from Cayman Chemical (Ann Arbor, MI, USA); nocodazole was from Sigma-Aldrich (St. Louis, MO, USA); Actinomycin D was obtained from Thermo Fisher Scientific. To induce EMT, cells were treated with 10 ng/mL TGFβ1 in completed medium containing 5% FBS.

### Plasmids and transfection

Complementary DNA (cDNA) of human SMAD2 (Genbank: BC014840) was obtained from transOMIC Technologies (Huntsville, AL, USA), amplified by PCR, and cloned into pcDNA3 vector (Thermo Fisher Scientific) with two HA tags inserted at the C-terminus of SMAD2 (i.e., SMAD2-HA). The nuclear-localizing green fluorescence protein (i.e., GFP-NLS) was constructed by inserting nuclear localization sequence (nucleotide sequence: 5ʹ-ccaaagaagaaacgcaaagtg-3ʹ; protein sequence: PKKKRKV) of SV40 Large T-antigen into 3ʹ end of pEGFP-C2 (Clontech). The expression vector of CFP-H2B (pH2b-CyFP) and YFP-lamin B1 (pYFP-laminB1) were sourced from Jan Ellenberg [62]. The mCherry-H2B expression vector was modified from pH2b-CyFP by replacing CFP with mCherry cDNA. The mCherry-lamin A expression vector was obtained by cloning full-length lamin A into pZome-1-C vector with mCherry at 5ʹ end driven by a CMV (cytomegalovirus) promoter. The GFP-lamin A expression vector was constructed by cloning GFP at the N-terminus of lamin A in pcDNA3 vector. The Lipofectamine™ 2000 (Thermo Fisher Scientific) transfection reagent was used to deliver the expression plasmids into cells in accordance with the protocol provided by the manufacturer.

### siRNAs and transfection

Sequences and/or manufacturers of the small interfering RNAs (siRNAs) used to deplete the expression of the targeted genes are listed in Additional file 1: Table S5. Cells were transfected with siRNAs via Lipofectamine™ RNAiMAX (Thermo Fisher Scientific) in accordance with the manufacturer’s protocol.

### RNA extraction and real-time quantitative PCR (qRT-PCR)

Total mRNAs were isolated from cells using RNeasy mini kit (Qiagen, Hilden, Germany). Complementary DNAs were produced using the SuperScript^®^ IV Reverse Transcriptase system (Thermo Fisher Scientific). qRT-PCR was carried out using Power SYBR Green master mix (Thermo Fisher Scientific). The qRT-PCR primers of H3.3 (i.e., *H3-3A* gene) was obtained from Qiagen (Cat. No. QT00247128). Gene expression levels were normalized to *GAPDH* using primers (forward: 5ʹ-GGAAGGTGAAGGTCGGAGTCA-3ʹ and reverse: 5ʹ-GTCATTGATGGCAACAATATCCACT-3ʹ).

### Immunoblotting

Expression of proteins in cells were analyzed by Western blotting against specific antibodies summarized in Additional file 1: Table S3. Cells were lysed with RIPA buffer [50 mM HEPES, pH 7.3, 150 mM NaCl, 2 mM EDTA, 20 mM β-gylcerophosphate, 0.1 mM Na_3_VO_4_, 1 mM NaF, 0.5 mM DTT and protease inhibitor cocktail (Roche Applied Science, Indianapolis, IN, USA)] containing 0.5% NP-40 with mild sonication to extract nuclear envelope and chromatin proteins. Total cell lysates were further lysed in 1× SDS sample buffer containing β-mercaptoethanol, analyzed by SDS-PAGE, transferred to polyvinylidene fluoride (PVDF, Millipore) membranes, and blotted with primary antibodies. Corresponding horse radish peroxidase (HRP) or alkaline phosphatase (AP)-conjugated secondary antibodies (Sigma-Aldrich) were added, and the blots were developed by chemiluminescence in accordance with the manufacturer’s protocols.

### Immunofluorescence staining and confocal microscopy

Cells were fixed in 4% paraformaldehyde for 30 min at room temperature and permeabilized with 0.5% Triton X-100 in phosphate buffered saline (PBS) for 30 min. For the immunofluorescence staining of histone H3.3, antigen retrieval was carried out by incubation in 100 °C citrate buffer (10 mM Citric Acid, pH 6.0) for 1 h, followed by incubation in 1% Triton X-100/PBS for 20 min. After two washes with PBS, cells were applied with 1% bovine serum albumin (BSA, Sigma-Aldrich)/PBS for 30 min at room temperature to block non-specific bindings. Then cells were incubated with primary antibodies (Additional file 1: Table S4) diluted in PBS for 1.5 h at room temperature. Fluorescent (Alexa-488, Alexa-568 or Alexa-633)-conjugated secondary antibodies (Thermo Fisher Scientific) at dilution 1/1000 were used for detection. For PLA experiments, cells seeded in 8-well chamber slides (Millicell EZ SLIDE, Millipore) were fixed with 4% paraformaldehyde in PBS for 15 min. Cells were permeabilized with 0.5% Triton X-100 in PBS for 30 min, and blocked with the Duolink^®^ Blocking Solution for 1 h at 37 °C. Primary antibodies diluted in Duolink^®^ Antibody Diluent where applied, and the slide were incubated for 1.5 h at room temperature. Detection of protein interactions was performed by following the manufacturer’s (Sigma-Aldrich) instructions. Cell nuclei were counterstained with Hoechst 33342 (Thermo Fisher Scientific) and mounted on slides using Prolong Gold antifade reagent (Thermo Fisher Scientific). Images were recorded using a Leica TCS SP5 confocal microscope (Leica, Wetzlar, Germany) equipped with HyD (hybrid detector). For live cell imaging, cells were incubated in a humidified chamber maintained at 37 °C and supplied with 5% CO_2_ (CU-109, Live Cell Instrument, Korea). Images were processed using Imaris 7.3 software (Bitplane, Zurich, Switzerland) and MetaMorph^®^ (Molecular Devices, San Jose, CA, USA).

### Immunogold staining and transmission electron microscopy (TEM)

Cells seeded on ACLAR^®^ film were fixed in a mixture containing 0.1% glutaraldehyde and 1% paraformaldehyde (Electron Microscopy Sciences, Hatfield, PA, USA) for 2 h on ice. Crosslinking was quenched using 0.125 M glycine, followed by neutralization with 0.1 M ammonium chloride. Cells were treated with a series of cold methanol dilutions, and then embedded in LR-Gold reagent on a Leica EM AFS2 (Leica Microsystems, Wetzlar, Germany). The embedded samples were stored in a humidity control box at room temperature. For immunogold labeling, ultrathin sections of LR-Gold embedded samples were mounted on 200 mesh nickel grids covered with carbon-backed formvar film. The grids were first incubated with 3% normal sheep serum in PBS at room temperature for 15 min, and incubated with a mouse anti-H1 antibody (sc-8030, Santa Cruz, Dallas, TX, USA) for 60 min. After 6 sequential washes with 1% normal sheep serum in PBS, the grids were incubated for 60 min with 18 nm gold–IgG complexes. The grids were washed sequentially with 1% normal sheep serum in PBS and 3% normal rat serum in PBS, followed by incubation with a rabbit anti-H3K27me3 antibody (#9733, Cell Signaling, Danvers, MA, USA) for 60 min. After another 6 sequential washes with 1% normal rat serum in PBS, the grids were incubated for 60 min with 12 nm gold–IgG complexes. Following another sequential wash with 1% normal rat serum in PBS, the grids were incubated with 3% normal donkey serum in PBS at room temperature for 15 min, and incubated with a goat anti-Lamin B antibody (sc-6217, Santa Cruz) for 60 min. After washes with 1% normal donkey serum in PBS, the grids were incubated for 60 min with 6 nm gold–IgG complexes. The grids were then washed sequentially with 1% normal donkey serum in PBS, followed by two washes with triple distilled water. Finally, the grids were treated with 2% uranyl acetate and 30 mM lead citrate. The final immunogold labeled grids were examined and photographed using a FEI Tecnai T12 electron microscope.

### Mass spectrometry

Spots excised from the Coomassie blue-stained SDS-PAGE were digested using MS grade Trypsin Gold (Promega, Madison, WI) overnight at 37 °C. The tryptic digests were extracted using 10 μL Milli Q water initially, followed by two extractions using a total of 20 μL 50% acetonitrile/0.1% trifluoroacetic acid. The combined extracts were dried in a vacuum concentrator, and then dissolved in 1 μL of 5% acetonitrile/0.5% trifluoroacetic acid. A Thermo Scientific™ Orbitrap Fusion™ Lumos™ Tribrid™ Mass Spectrometer (Thermo Fisher Scientific) was used to detect electrospray ionization (ESI)–MS/MS and higher energy collisional dissociation (HCD)–MS/MS peptide signals. The MS/MS signal was analyzed using the MASCOT search engine (www.matrixscience.com).

### Calculation of axial ratio (AR)

Nuclear morphology was quantified by calculating the axial ratio (AR) of each nucleus, determined from the nuclear staining of Hoechst 33342 cells. A custom Matlab code was developed to trace the nuclear perimeter, and applied elliptical Fourier analysis (EFA) to find the first 20 elliptic harmonics [23]. AR was defined as the sum of the axes from the first 20 ellipses normalized by the first ellipse, subtracted by one. AR represents deviations from a perfect elliptical shape, where bigger ellipses from the later harmonics would result in a larger AR value.

### Statistical analysis

Data and statistical analyses were performed using Microsoft Excel and Graphpad Prism software. Data were analyzed using two-tailed Student’s *t* test or Fisher’s exact test. *P* values below 0.05 were considered significant.

## Supplementary Information

**Additional file 1.** Supplementary Tables and Figures.

**Additional file 2: Movie S1.** Live-cell confocal imaging of the nucleus (denoted by mCherry-H2B) in Huh7 cells.

**Additional file 3: Movie S2.** Live-cell confocal imaging of the nucleus (denoted by mCherry-H2B) in Huh7 cells treated with TGFβ1.

**Additional file 4: Movie S3.** Live-cell confocal imaging of GFP-lamin A (green) and mCherry-H2B (red) in Huh7 cells treated with TGFβ1.

**Additional file 5: Movie S4A.** Live-cell confocal imaging of YFP-lamin B1 (green) and mCherry-lamin A (red) in Huh7 cells treated with TGFβ1.

**Additional file 6: Movie S4B.** Live-cell confocal imaging of YFP-lamin B1 (green), mCherry-lamin A (red) and CFP-H2B (cyan) in Huh7 cells treated with TGFβ1.

**Additional file 7: Movie S5A.** Live-cell confocal imaging of GFP-NLS (green) and mCherry-lamin A (red) in Huh7 cells treated with TGFβ1.

**Additional file 8: Movie S5B.** Live-cell confocal imaging of GFP-NLS (green), mCherry-lamin A (red) and CFP-H2B (cyan) in Huh7 cells treated with TGFβ1.

**Additional file 9: Movie S6.** Live-cell confocal imaging of GFP-NLS and mCherry-H2B in LMNA_KO Huh7 cells treated with TGFβ1.

## Data Availability

All data generated or analyzed during this study are included in this published article (and its additional information files).
