# Supplementary material for: Deformation of the nucleus by TGFβ1 via the remodeling of nuclear envelope and histone isoforms
Source: Epigenetics Chromatin. 2022 Jan 4;15:1. doi: 10.1186/s13072-021-00434-3 (PMC8725468; doi:10.1186/s13072-021-00434-3)
Supplement: Supplementary file 1 — Additional file 1. Supplementary Tables and Figures. [file 13072_2021_434_MOESM1_ESM.docx]

**Deformation of the nucleus by TGFβ1 via the remodeling of nuclear envelope and histone isoforms**

Ya-Hui Chi^1,2*^, Wan-Ping Wang^1^, Ming-Chun Hung^1^, Gunn-Guang Liou^3^, Jing-Ya Wang^1^ and Pen-Hsiu Grace Chao^4^

^1^Institute of Biotechnology and Pharmaceutical Research, National Health Research Institutes, Zhunan, Miaoli County 35053, Taiwan; ^2^Graduate Institute of Biomedical Sciences, China Medical University, Taichung 40402, Taiwan; ^3^National Taiwan University College of Medicine, Taipei 10051, Taiwan; ^4^Department of Biomedical Engineering, School of Medicine and School of Engineering, National Taiwan University, Taipei 10617, Taiwan.

Running title: Nuclear shape linked to TGFβ1 signaling

Keywords: Nuclear envelope, nuclear lamina, nuclear morphology, TGFβ1

^*^ Corresponding author

Ya-Hui Chi

ORCID: 0000-0002-9216-0938

35 Keyan Road, Zhunan, Miaoli County 35053, Taiwan

Ph: +886-37-206166ext35718; Fax: +886-37-586456

Email: [ychi@nhri.edu.tw](mailto:ychi@nhri.edu.tw)

**Additional file 1: Supplementary Tables and Figures**

**Table S1** LC-MS/MS quantification for the post-translation modification of histone H3 in Huh7.

**Table S2** Relative abundance of histone variants in TGFβ1- versus mock- treated Huh7 cells.

**Table S3** List of antibodies used in Western blot analysis.

**Table S4** List of antibodies used in immunofluorescence staining.

**Table S5** List of siRNAs.

**Figure S1** TGFβ1 induces changes in nuclear morphology in multiple cell lines.

**Figure S2** SUN1 and lamin B1 contribute to TGFβ1-induced nuclear deformation.

**Figure S3** Expression and localization of NE proteins in TGFβ1-treated cells.

**Figure S4** TGFβ1-induced nuclear morphology change is a downstream process of SMAD signaling.

**Figure S5** Association between variants and epigenetic status of histones with NE rupture.

**Supplementary Tables**

**Table S1.** LC-MS/MS quantification for the post-translation modification of histone H3 in Huh7.

| **Treatment** | **Protein Name** | **PTM** | **PTM Sequence** | **PTM spectrum** |
| --- | --- | --- | --- | --- |
|  |  |  |  | **(Count)** |
| Mock | Histone H3.1 |  |  |  |
|  |  | Acetyl (K) | **Total Acetyl sequences** | 13 |
|  |  |  | K.STGGK#APR.K | 3 |
|  |  |  | R.KQLATK#AAR.K | 4 |
|  |  |  | R.K#SAPATGGVKKPHR.Y | 1 |
|  |  |  | R.RYQK#STELLIR.K | 3 |
|  |  |  | R.EIAQDFK#TDLR.F | 1 |
|  |  |  | R.VTIMPK#DIQLAR.R | 1 |
|  |  | Methyl (K) | **Total Methyl sequences** | 9 |
|  |  |  | R.K#SAPATGGVKKPHR.Y | 7 |
|  |  |  | K.SAPATGGVKK#PHR.Y | 1 |
|  |  |  | R.EIAQDFK#TDLR.F | 1 |
| TGFβ1 | Histone H3.3 |  |  |  |
|  |  | Acetyl (K) | **Total Acetyl sequences** | 5 |
|  |  |  | K.QLATK#AAR.K | 2 |
|  |  |  | R.K#SAPSTGGVK.K | 1 |
|  |  |  | R.EIAQDFK#TDLR.F | 2 |
|  |  | Methyl (K) | **Total Methyl sequences** | 2 |
|  |  |  | R.EIAQDFK#TDLR.F | 2 |

| **Table S2.** Relative abundance of histone variants in TGFβ1- versus mock- treated Huh7 cells. | | | | |
| --- | --- | --- | --- | --- |
| **Protein Name** | **Protein accession No.** | **Score Mascot** | **Protein coverage [%]** | **Relative abundance (TGFβ1/mock)** |
| Histone H1.1 | Q02539 | 67 | 7 | 1.786 |
| Histone H1.2 | P16403 | 67 | 13 | 1.331 |
| Histone H1.3 | P16402 | 67 | 12 | 1.331 |
| Histone H1.4 | P10412 | 67 | 12 | 1.331 |
| Histone H1t | P22492 | 67 | 7 | 1.786 |
| Histone H2A type 1-B/E | P04908 | 640 | 44 | 0.250 |
| Histone H2A type 1-D | P20671 | 922 | 44 | 0.635 |
| Histone H2A type 2-B | Q8IUE6 | 603 | 44 | 0.404 |
| Histone H2A type 3 | Q7L7L0 | 640 | 44 | 0.250 |
| Histone H2A.V | Q71UI9 | 222 | 31 | 0.756 |
| Histone H2A.Z | P0C0S5 | 222 | 31 | 0.756 |
| Histone H2AX | P16104 | 687 | 45 | 1.105 |
| Histone H2B type 1-A | Q96A08 | 768 | 43 | 1.245 |
| Histone H2B type 1-B | P33778 | 3524 | 83 | 1.336 |
| Histone H2B type 1-C/E/F/G/I | P62807 | 3633 | 75 | 1.699 |
| Histone H2B type 1-D | P58876 | 3633 | 75 | 1.699 |
| Histone H2B type 1-H | Q93079 | 3633 | 75 | 1.699 |
| Histone H2B type 1-L | Q99880 | 3495 | 75 | 1.245 |
| Histone H2B type 1-M | Q99879 | 3633 | 83 | 1.699 |
| Histone H2B type 1-O | P23527 | 3523 | 75 | 1.336 |
| Histone H2B type 2-E | Q16778 | 3523 | 75 | 1.336 |
| Histone H2B type 2-F | Q5QNW6 | 3633 | 75 | 1.699 |
| Histone H3.1 | P68431 | 2536 | 89 | 0.984 |
| Histone H3.1t | Q16695 | 2183 | 63 | 0.901 |
| Histone H3.3 | P84243 | 2207 | 89 | 2.571 |
| Histone H3.3C | Q6NXT2 | 1265 | 45 | 1.153 |
| Histone H3.Y | P0DPK2 | 563 | 12 | 1.307 |
| Histone H3-like centromeric protein A | P49450 | 42 | 19 | 2.170 |
| Histone H4 | P62805 | 1450 | 62 | 0.354 |

**Table S3**. List of antibodies used in Western blot analysis.

| Antibody | Make | Catalog No. | Dilution | Species |
| --- | --- | --- | --- | --- |
| β-Catenin | Cell Signaling | #8480 | 1/2000 | rabbit |
| N-Cadherin | Cell Signaling | #13116 | 1/1000 | rabbit |
| Vimentin | Abcam | ab92547 | 1/10,000 | rabbit |
| β-ACTIN | Sigma-Aldrich | A1978 | 1/100,000 | mouse |
| SMAD3 | Abcam | ab40854 | 1/10,000 | rabbit |
| p-SMAD3 (Ser423+Ser425) | Abcam | ab52903 | 1/2,000 | rabbit |
| SMAD2 | Abcam | ab40855 | 1/2,000 | rabbit |
| Lamin A | Millipore | MAB3540 | 1/5,000 | mouse |
| Lamin A/C | Abcam | ab108595 | 1/5,000 | rabbit |
| Lamin B1 | Abcam | ab133741 | 1/10,000 | rabbit |
| SUN1 | Sourced from NIH (*13*) |  | 1/5,000 | rabbit |
| SUN2 | Epitomics | 5279-1 | 1/2,000 | rabbit |
| Emerin | Santa Cruz | Sc-25284 | 1/5,000 | mouse |
| Histone H3 | Millipore | 07-690 | 1/250,000 | rabbit |
| Histone H3.3 | Abcam | ab176840 | 1/5,000 | rabbit |
| Histone H1.2 | Abcam | ab181973 | 1/2,000 | rabbit |
| Histone H1.3 | Abcam | ab24174 | 1/1,000 | rabbit |
| Histone H1.4 | Invitrogen | 703551 | 1/1,000 | rabbit |
| Histone H1.5 | Abcam | ab18208 | 1/2,000 | rabbit |
| H3K27me3 | Cell Signaling | #9733 | 1/2,000 | rabbit |

**Table S4**. List of antibodies used in immunofluorescence staining.

| Antibody | Make | Catalog No. | Dilution | Species |
| --- | --- | --- | --- | --- |
| Lamin B | Santa Cruz | sc-6217 | 1/300 | goat |
| Lamin A | Millipore | MAB3540 | 1/300 | mouse |
| Lamin A/C | Abcam | ab108595 | 1/500 | rabbit |
| H3K27me3 | Abcam | ab6002 | 1/200 | mouse |
| H3K27me3 | Cell Signaling | #9733 | 1/1,000 | rabbit |
| Histone H1 | Santa Cruz | sc-8030 | 1/600 | mouse |
| Histone H3.3 | Abcam | ab183902 | 1/500 | rabbit |
| HA | Sigma-Aldrich | H6908 | 1/1,000 | rabbit |
| SUN1 | Sourced from NIH (*13*) |  | 1/300 | rabbit |
| NPC | Sigma-Aldrich | N8786 | 1/500 | mouse |
| Emerin | Santa Cruz | sc-25284 | 1/100 | mouse |
| Vimentin | Millipore | AB5733 | 1/1500 | chicken |
| α-tubulin | Sigma-Aldrich | T5168 | 1/1500 | mouse |
| Phalloidin | Invitrogen | A12379 | 1/200 | N/A |
| H3K4me3 | Cell Signaling | #9751 | 1/400 | rabbit |
| H3K9me3 | Abcam | ab8898 | 1/1,000 | rabbit |
| H3K27me1 | Abcam | ab194688 | 1/100 | rabbit |
| H3K27me2 | Cell Signaling | #9728 | 1/3,000 | rabbit |
| H3K18ace | Cell Signaling | #13998 | 1/200 | rabbit |
| H4K16ace | Millipore | 07-329 | 1/500 | rabbit |

**Table S5**. List of siRNAs.

| Gene | Target sequence | Make | Catalog No. |
| --- | --- | --- | --- |
| *LMNA* | AACTGGACTTCCAGAAGAACA | Qiagen | SI02654862 |
| *LMNB1* | AACGCGCTTGGTAGAGGTGGA | Qiagen | SI00300671 |
| *SUN1* | CCATCCTGAGTATACCTGTCTGTAT | Invitrogen | Hs_Sun1-2301 |
| *SUN2* | AACCAAAGCAGGGTGGAATGTCTGC  TGAAGAGGTCTTCTGAGTCTTGCTG | Invitrogen | Hs_Sun2-714  Hs_Sun2-1099 |
| *EMD (Emerin)* | GCCTCCTCTTATAGCTTCTCTGACT | Invitrogen | HSS103213 |
| *H1-4 (H1.4)* | N/A | Ambion | s6402 |
| *H1-5 (H1.5)* | N/A | Ambion | s6405 |
| *H1-2 (H1.2)* | N/A | Ambion | s6397 |
| *H1-3 (H1.3)* | N/A | Ambion | s6398 |
| *H3-3A (H3.3)* | CAGCGGTTCAACTTTATAATA  ATACGTGGAGAACGTGCTTAA | Qiagen | SI04357514  SI04338152 |
| *SMAD2* | CAGGTAATGTATCATGATCCA  AAGCCGTCTATCAGCTAACTA | Qiagen | SI02757496  SI03033275 |
| *SMAD3* | AGCCTATACTTTGGCAGGTTA  AAGGAGCACCTTGACAGACTT  AAGAGATTCGAATGACGGTAA  ATCAAGGGATTTCCTATGGAA | Qiagen | SI05062645  SI00082502  SI00082495  SI00082481 |

N/A: not available

**Supplementary Figures**

**
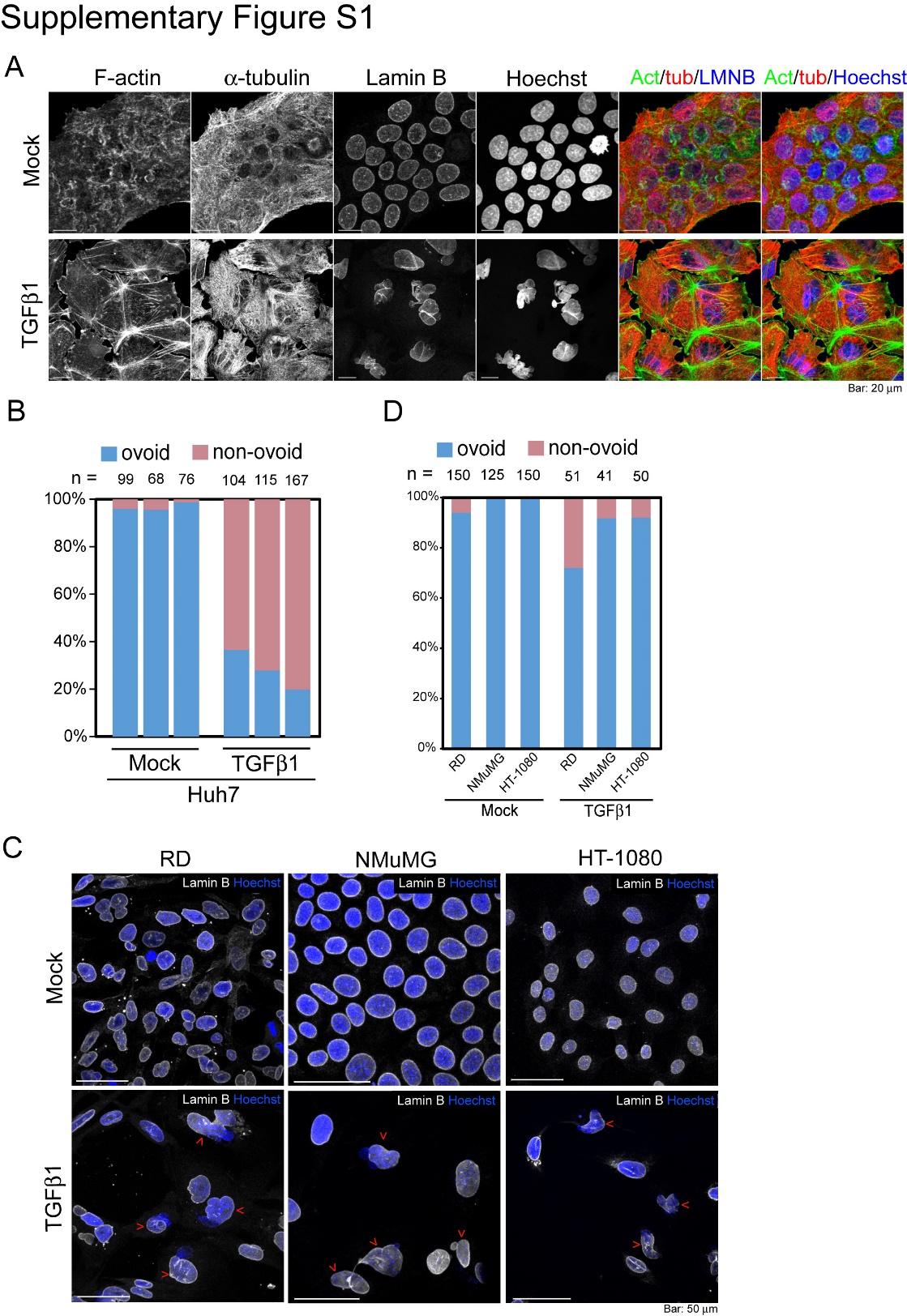
**

**Figure S1****. TGFβ1 induces changes in nuclear morphology in multiple cell lines.** (A) Confocal images of Huh7 cells with mock or with TGFβ1 treatment for 48 h. Cells were immunofluorescent stained with phalloidin (to denote F-Actin), mouse anti-α-tubulin and goat anti-lamin B. Nuclei were counterstained with Hoechst 33342. Act, F-actin; tub, α-tubulin, LMNB, lamin B. (B) Characterization of the morphology (ovoid or non-ovoid) of cells treated with TGFβ1 or mock-treated. Number of cells quantified in experiments performed in triplicate was denoted. *P* < 0.0001 comparing mock- and TGFβ1-treated cells, Fisher’s exact test. (C) Nuclear morphology of RD (human rhabdomyosarcoma), NMuMG (mouse mammary gland epithelial cell), and HT-1080 (fibrosarcoma) cells treated with 10 ng/mL TGFβ1 or mock-treated for 3 days. The red arrow heads denote cells with deformed nuclei. Cells were fixed using 4% paraformaldehyde and subjected to immunofluorescence staining with lamin B (white). The nuclei were counter stained with Hoechst 33342 (blue). (D) Quantification for the morphology (ovoid or non-ovoid) of RD, NMuMG and HT-1080 as illustrated in (C). Number of cells quantified under each experiment condition was denoted. All images are the sum of z-stacks.

**
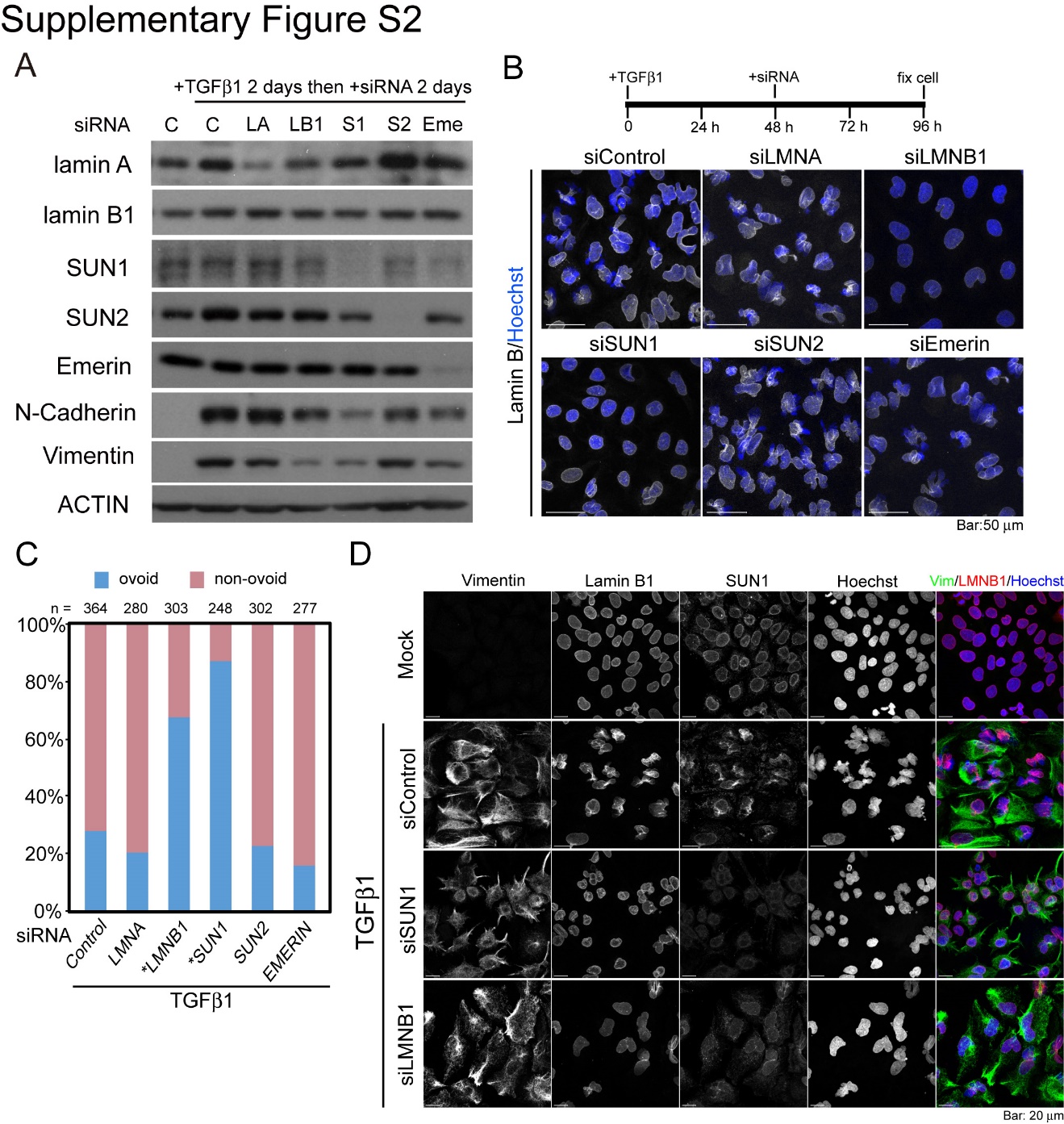
**

**Figure S2.** **SUN1 and lamin B1 contribute to TGFβ1-induced nuclear deformation.** (A) Immunoblotting results of the indicated proteins in Huh7 cells first treated with TGFβ1 for two days, then transfected with the indicated siRNAs for another two days. (B) Cells were treated using the upper scheme, and were immunofluorescent stained with anti-lamin B (white). Nuclei were counterstained with Hoechst 33342 (blue). Images are the sum of z-stacks. (C) Quantification for the morphology (ovoid or non-ovoid) of cells treated using the methods in (B). Number of cells quantified under each experiment condition was denoted. *, *P* < 0.0001, Fisher’s exact test. (D) Cells were treated as described in the scheme of (B), and were immunofluorescent stained with chicken anti-Vimentin, goat anti-lamin B, and rabbit anti-SUN1. Nuclei were counterstained with Hoechst 33342. Images are the sum of z-stacks.

**
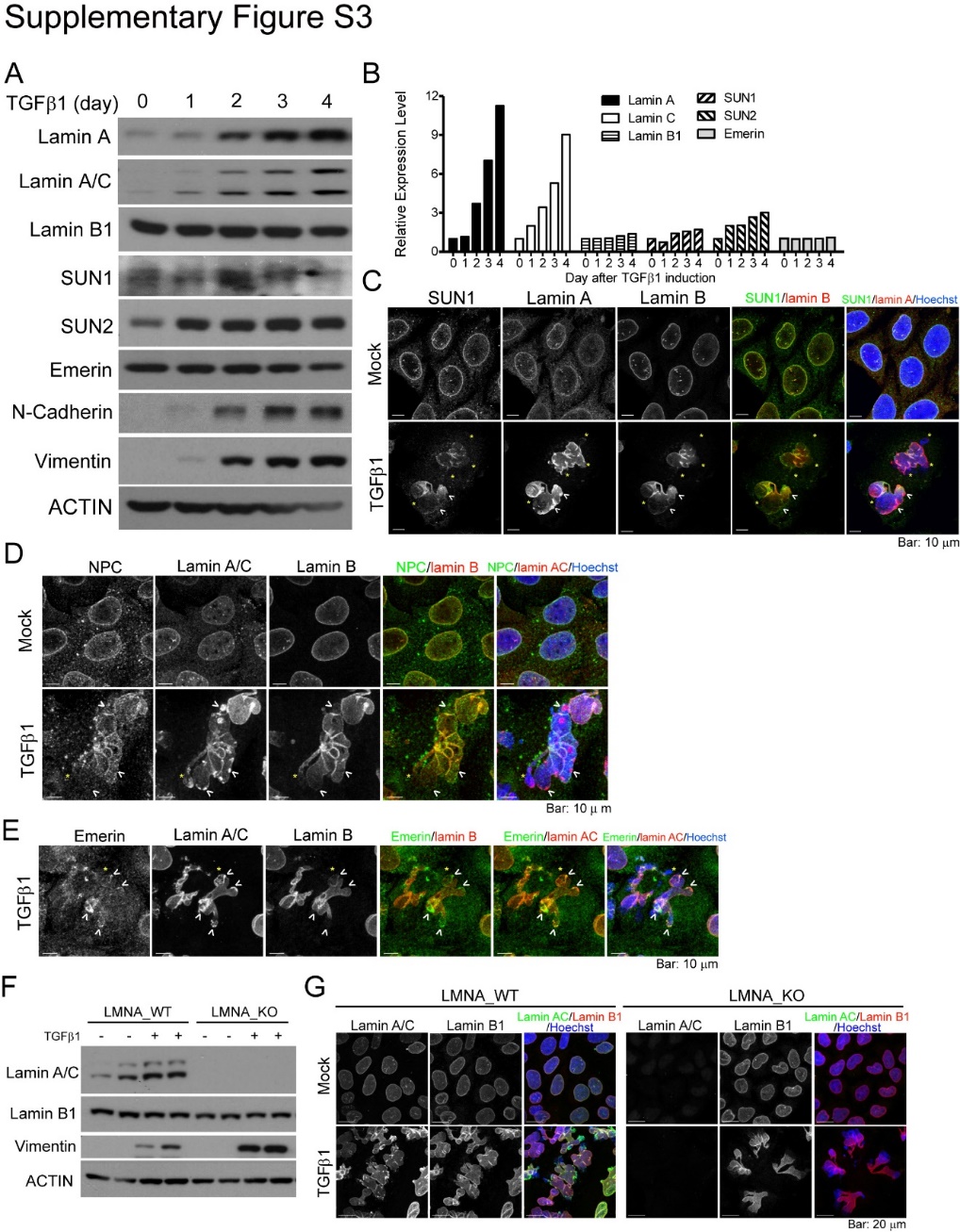
**

**Figure S3.** **Expression and localization of NE proteins in TGFβ1-treated cells.** (A) Western blot analysis for the protein expression of nuclear lamins, NE proteins, and mesenchymal markers N-Cadherin and Vimentin in Huh7 cells harvested after TGFβ1 treatment for 0-4 days. ACTIN was used as a loading control. (B) Quantification of protein expression levels presented in (A). (C-E) Immunofluorescence staining images of Huh7 cells mock- or TGFβ1-treated for 2 days. The primary antibodies were rabbit anti-SUN1, mouse anti-lamin A, goat anti-lamin B, mouse anti-nuclear pore complex (NPC, clone mAb414), rabbit anti-lamin A/C and mouse anti-Emerin. The yellow star indicates the NE stained negative for both lamin A and lamin B. The white arrow head indicates the NE stained positive for lamin A and negative for lamin B. Nuclei were counterstained using Hoechst 33342. (F) Western blot analysis for the expression of nuclear lamins and Vimentin in LMNA_WT and LMNA_KO Huh7 cells with/without TGFβ1 treatment for 72 h. Vimentin was used as a marker of EMT, and ACTIN is a loading control. (G) Confocal imaging of LMNA_WT and LMNA_KO Huh7 cells treated mock or TGFβ1 for 48 h, and immunofluorescent stained with lamin A/C and lamin B antibodies. Nuclei were counter stained with Hoechst 33342.

**
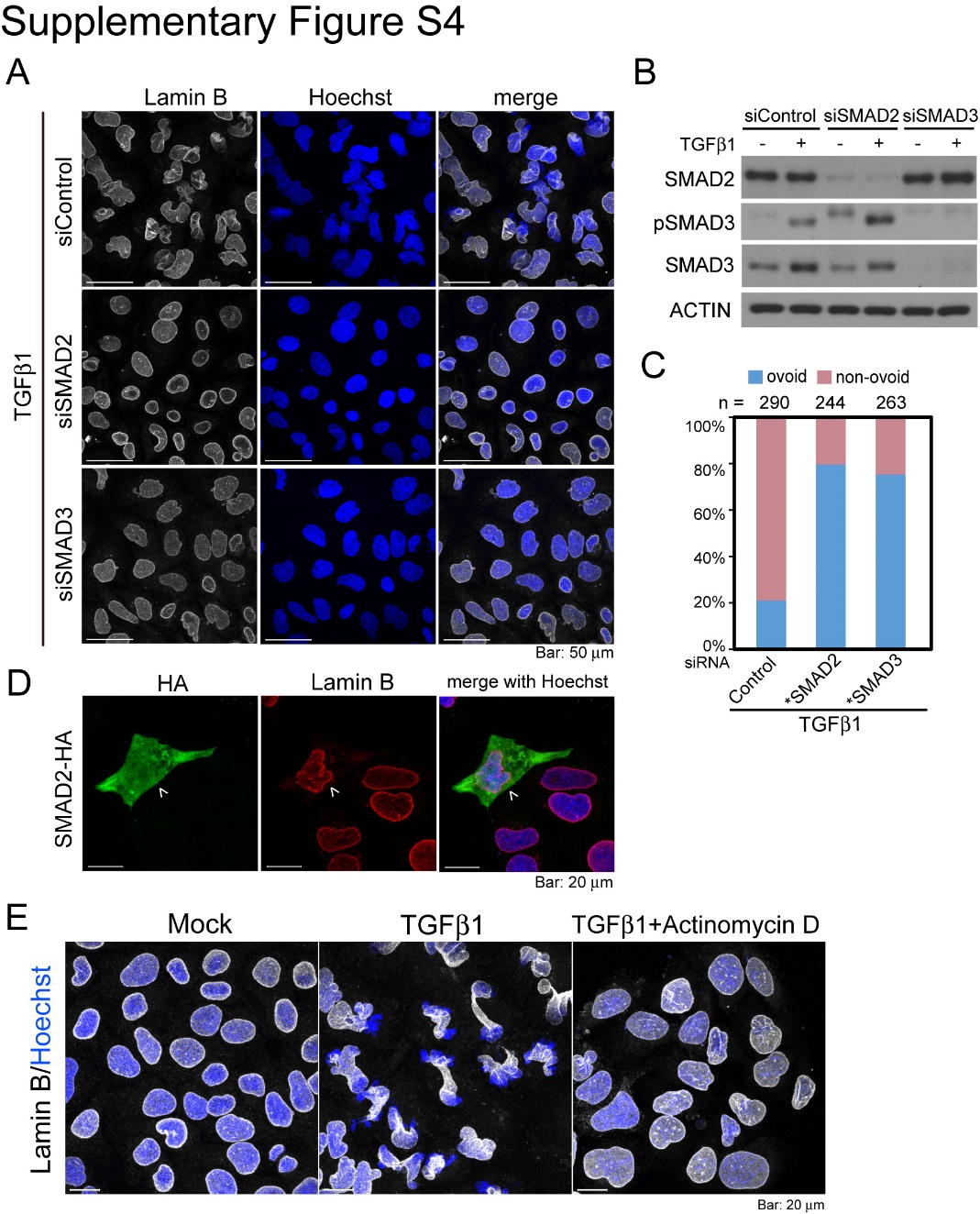
**

**Figure S4.** **TGFβ1-induced nuclear morphology change is a downstream process of SMAD signaling.** (A) Confocal images of the nucleus in control, SMAD2 or SMAD3 siRNA-treated cells induced to undergo EMT by TGFβ1. Cells were fixed after 48 h of TGFβ1 treatment, and immunofluorescent stained using goat anti-lamin B (white). Nuclei were counter stained with Hoechst 33342 (blue). Images are the sum of z-stacks. (B) Western blot analysis for SMAD2 and SMAD3 in Huh7 cells treated using the methods in (A). ACTIN immunoblotting was used as a loading control. (C) Quantification of changes in the nuclear morphology (ovoid or non-ovoid) of cells treated with siRNAs respectively targeting SMAD2 and SMAD3, and induced to undergo EMT by TGFβ1 for 48 h. A total of 250-350 cells were quantified in each experiment. *, *P* < 0.0001, Fisher’s exact test. (D) Confocal images of Huh7 cells transfected with a plasmid expressing HA-tagged SMAD2 (SMAD2-HA), and immunofluorescent stained with anti-HA (green) and anti-lamin B (red) antibodies. The white arrow head denotes a cell expressing SMAD2-HA. The nuclear morphology of the cell expressing SMAD2-HA is deformed (non-ovoid), whereas the un-expressed cells present an ovoid nuclear morphology. Nuclei were counter stained using Hoechst 33342 (blue). Images are the sum of z-stacks. (E) Confocal images of mock or TGFβ1-treated Huh7 cells co-treated with/without 100 ng/mL of Actinomycin D for 48 h, and immunofluorescent stained using goat anti-lamin B (white). Nuclei were counter stained with Hoechst 33342 (blue). Images are the sum of z-stacks.

**
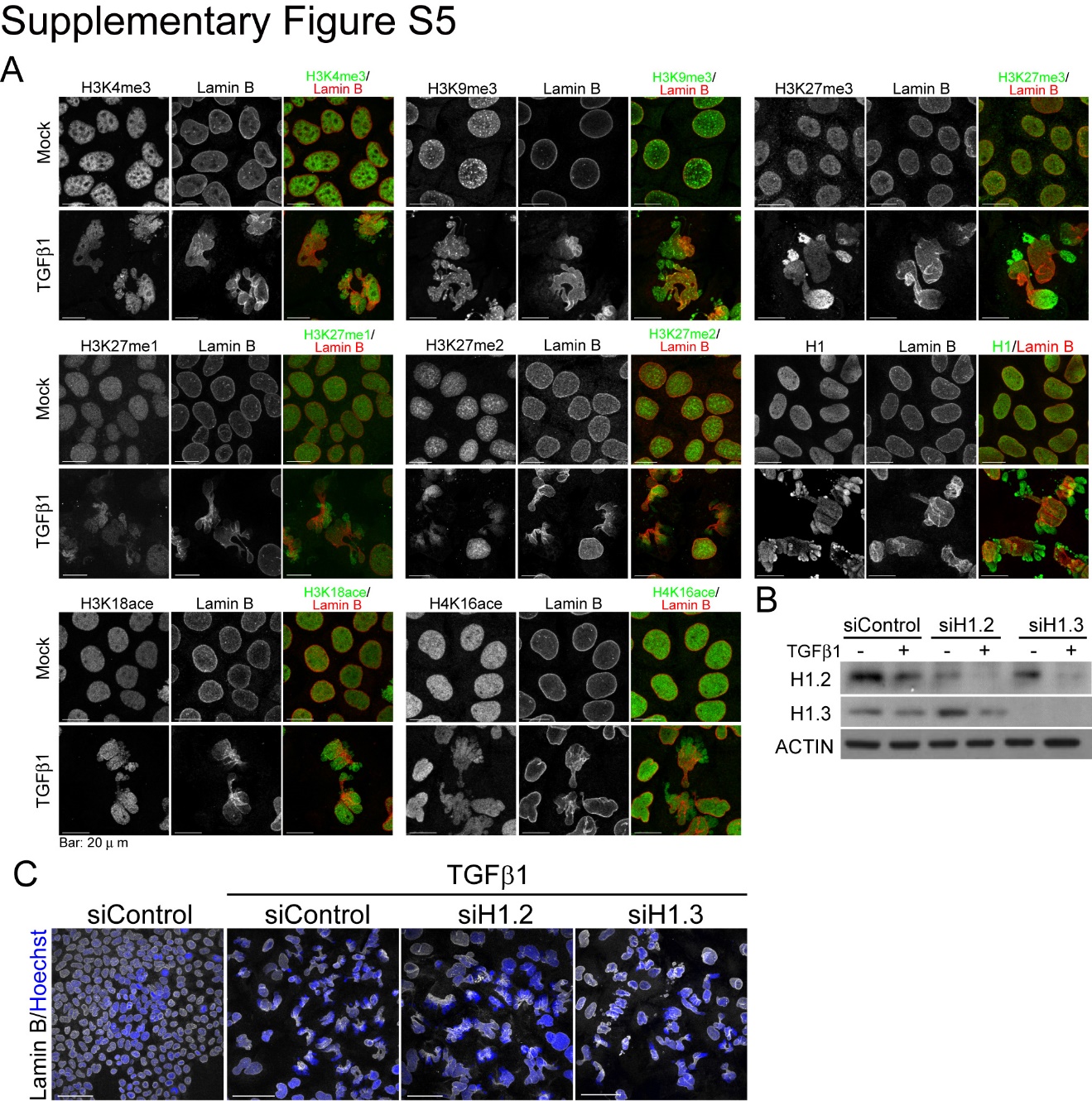
**

**Figure S5.** **Association between variants and epigenetic status of histones with NE rupture.** (A) Relative distribution of the epigenetically modified histones and lamin B in mock- and TGFβ1-treated cells for 48 h. Cells were immunofluorescent stained using the indicated antibodies. Images are the sum of z-stacks. (B) Western blot analysis indicating the knocking down efficiency of H1.2 and H1.3 by siRNAs in Huh7 cells, followed by mock- or TGFβ1-treatment for 48 h. (C) Morphology of nucleus in Huh7 cells depleted for H1.2 or H1.3, followed by TGFβ1 treatment for 48 h. Cells were immunofluorescent stained with goat anti-lamin B (white). Nuclei were counterstained with Hoechst 33342 (blue). Images are the sum of z-stacks. Scale bars: 50 μm.
